# Controlled oligomerization of [1.1.1]propellane through radical polarity matching: selective synthesis of SF_5_- and CF_3_SF_4_-containing [2]staffanes

**DOI:** 10.3762/bjoc.20.259

**Published:** 2024-11-29

**Authors:** Jón Atiba Buldt, Wang-Yeuk Kong, Yannick Kraemer, Masiel M Belsuzarri, Ansh Hiten Patel, James C Fettinger, Dean J Tantillo, Cody Ross Pitts

**Affiliations:** 1 Department of Chemistry, University of California, Davis, 1 Shields Avenue, Davis, CA 95616, U.S.A.

**Keywords:** pentafluorosulfanylation, [1.1.1]propellane, radical chain oligomerization, staffanes, strain-release

## Abstract

Selectivity in radical chain oligomerizations involving [1.1.1]propellane – i.e., to make [*n*]staffanes – has been notoriously challenging to control when *n* > 1 is desired. Herein, we report selective syntheses of SF_5_- and CF_3_SF_4_-containing [2]staffanes from SF_5_Cl and CF_3_SF_4_Cl, demonstrating cases whereby oligomerization is preferentially truncated after incorporation of two bicyclopentane (BCP) units. Synthetic and computational studies suggest this phenomenon can be attributed to alternating radical polarity matching. In addition, single-crystal X-ray diffraction (SC-XRD) data reveal structurally interesting features of the CF_3_SF_4_-containing [2]staffane in the solid state.

## Introduction

In various radical additions of X–Y across [1.1.1]propellane (**1**), functionalized oligomers known as [*n*]staffanes – with *n* > 1, where *n* denotes the number of individual [1.1.1]bicyclopentane (BCP) linkers – are often observed and swiftly devalorized as *side-products* [1]. However, targeted synthesis of functionalized [*n*]staffanes as rigid "molecular spacers" as proposed by Kaszynski and Michl [2–4] could facilitate new developments in nanotechnology [5], liquid crystal design [6–10], and the study of energy-transfer [11–12] or electron-transfer [13–17] processes. We also posit that lower-order [*n*]staffanes (i.e., *n* = 2 or 3) are potentially valuable C(sp^3^)-rich bioisosteres [18–19] that have been seemingly overlooked in the medicinal chemistry arena, in stark contrast to single BCP units over the past 12 years [20–24].

**Figure 1:**
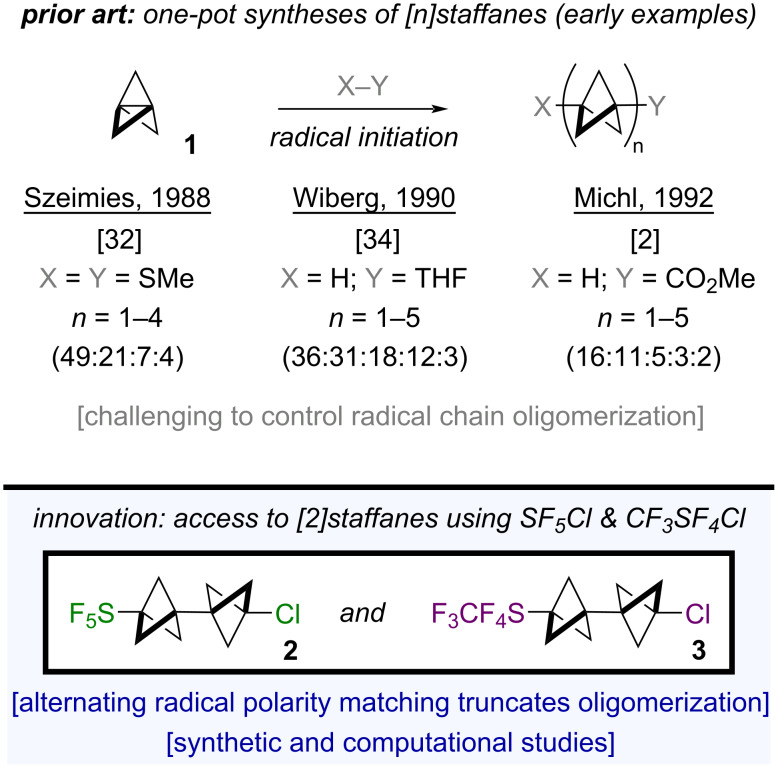
(Top) highlighting selectivity challenges in the synthesis of [*n*]staffanes using excess [1.1.1]propellane (**1**). (Bottom) selective synthesis of [2]staffanes bearing the SF_5_ (**2**) and CF_3_SF_4_ (**3**) groups (this work).

One plausible explanation for the paucity of applications of [*n*]staffanes in materials or biological settings is a synthetic accessibility issue. For instance, dimerization of substituted BCPs [25–27] or photochemical appendage of **1** onto an extant BCP [28–31] are relatively effective tactics for the selective assembly of certain [*n*]staffanes; the main caveat is that multiple synthetic steps are required. On the other hand, while a one-pot radical chain oligomerization is conceptually appealing, radical additions of X–Y across **1** in practice can be challenging to control and often lead to complex mixtures of functionalized [*n*]staffanes, *n* = 1–5 (Figure 1, top) [2,31–34]. Even though [*n*]staffanes are often separable by column chromatography, the yields for a single oligomer across a panoply of different transformations typically range from <1% to ≈30% when *n* > 1 is desired [35]. To the best of our knowledge, the assembly of functionalized [*n*]staffanes from **1** in high yield/selectivity and in one step via controlled radical oligomerization remains a synthetic challenge.

Herein, we report proof-of-concept that our previous work on strain-release pentafluorosulfanylation of **1** [36] using SF_5_Cl (prepared in house [37] under mild oxidative fluorination conditions [38–44]) can be extended to the selective synthesis of the associated chloropentafluorosulfanylated [2]staffane (SF_5_-BCP-BCP-Cl, **2**), based on alternating radical polarity matching in the chain-propagation steps (Figure 1, bottom) [45–47]. Density functional theory (DFT) calculations provide insight into our observed selectivity, and our hypothesis is bolstered by computation of relative bicyclopentyl radical philicities. In addition, we demonstrate that similar reaction conditions can be applied to the synthesis of the analogous CF_3_SF_4_-containing [2]staffane (CF_3_SF_4_-BCP-BCP-Cl, **3**). Finally, we examined compound **3** by SC-XRD and found that it undergoes a phase transition as a function of rate of cooling; this highlights that the [2]staffanes synthesized during this study are also interesting from a fundamental structural standpoint.

## Results and Discussion

Over the past few years, our group has begun to establish strain-release pentafluorosulfanylation as a viable strategy for C(sp^3^)–SF_5_ bond formation [35,48]. For instance, in 2022, we reported a method for chloropentafluorosulfanylation of [1.1.1]propellane, i.e., to make SF_5_-BCP-Cl (**4**), that ostensibly proceeds through a radical chain propagation mechanism [36]. Under optimized conditions, we obtained product **4** in 86% yield, and the corresponding [2]staffane – SF_5_-BCP-BCP-Cl (**2**) – was formed as a minor side-product in 7% yield. While our original goal was to suppress formation of **2**, we later pondered whether preferential synthesis of compound **2** would also be possible. Accordingly, we began our screening process by systematically increasing the equivalents of [1.1.1]propellane (**1**) relative to SF_5_Cl and evaluating the impact on selectivity (Table 1).

**Table 1:** Effect of [1.1.1]propellane (**1**) equivalents relative to SF_5_Cl on selectivity^a^.

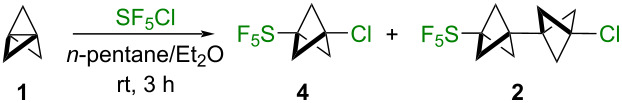				

entry	**1** (equiv)	**4** (% yield)^b^	**2** (% yield)^b^	**4**:**2**

1	1.0	49%	4%	12:1.0
2	2.0	74%	22%	3.4:1.0
3	3.0	44%	29%	1.5:1.0
4	4.0	54%	40%	1.3:1.0
5	6.0	48%	45%	1.1:1.0
6	8.0	43%	53%	1.0:1.3
7	10	32%	51%	1.0:1.6
8	20	24%	72%	1.0:3.0
**9** **^c^**	**6.0**	**30%**	**63% (51%)** **^d^**	**1.0:2.1**

^a^A 0.1 M solution of SF_5_Cl in *n*-pentane (0.1 mmol) was added to a 0.8 M solution of [1.1.1]propellane in Et_2_O under Ar atmosphere and stirred at rt for 3 h. ^b^Yield determined by ^19^F NMR; ^c^SF_5_Cl was added portion-wise. ^d^Isolated yield.

A 0.1 M solution of SF_5_Cl in *n*-pentane was added to a 0.8 M solution of **1** in Et_2_O at room temperature, and the mixture was stirred for 3 hours prior to ^19^F NMR analysis. Upon increasing from 1.0 to 6.0 equiv of **1**, we observed the **4**:**2** product ratio decreased dramatically from 12:1 to 1.1:1. Using 8.0 equiv of **1**, the ratio flipped such that **2** became the major product (i.e., **4**:**2** = 1:1.3) and was formed in 53% yield. Interestingly, even with 8.0 equiv of **1**, 96% of the material balance could be accounted for in the formation of *these two products alone* by ^19^F NMR. To the best of our knowledge, this is an exceptionally rare instance whereby oligomerization of **1** appears to be stunted beyond formation of the [2]staffane; only trace yields of putative higher-order staffanes (*n* > 2) were detected in the crude reaction mixture. Remarkably, using 20 equiv of **1**, the observed **4**:**2** product ratio improved to 1:3, with **2** formed in 72% yield. In an even more extreme case, the [2]staffane still remained the major product formed in 66% yield when using 50 equiv of **1** (^19^F NMR signals of presumed higher-order staffanes became more apparent, though their combined yield still remained ≈18%).

Ultimately, we found adding SF_5_Cl portion-wise – to keep the "effective" equiv of **1** higher at any given moment – proved to be a suitable compromise to access **2** in 63% yield by ^19^F NMR (51% isolated) using 6.0 equiv of **1** (Table 1). We also demonstrated that this procedure can be performed on a 4.0 mmol scale (with respect to SF_5_Cl) to provide access to ≈0.5 g of **2** in 43% isolated yield. Additional details on reaction optimization can be found in Supporting Information File 1.

Upon increasing the equivalents of **1** during the screening process, we also found that irradiation with white LEDs was not necessary to boost product yields, as it was in our previously reported synthesis of **4** [36]. Both our laboratory [36] and the Qing laboratory [49] have previously observed that SF_5_Cl additions to **1** can proceed in the absence of light. Note that recent work from the laboratories of Cahard and Bizet [50] suggests that autoxidation of the ethereal solvent could serve as one possible explanation for initial formation of SF_5_ radicals in the absence of light to initiate a radical chain reaction. It is also well established that [1.1.1]propellane participates in radical-chain reactions (i.e., oligomerization) at room temperature in solution to form unsubstituted [*n*]staffanes.

**Figure 2:**
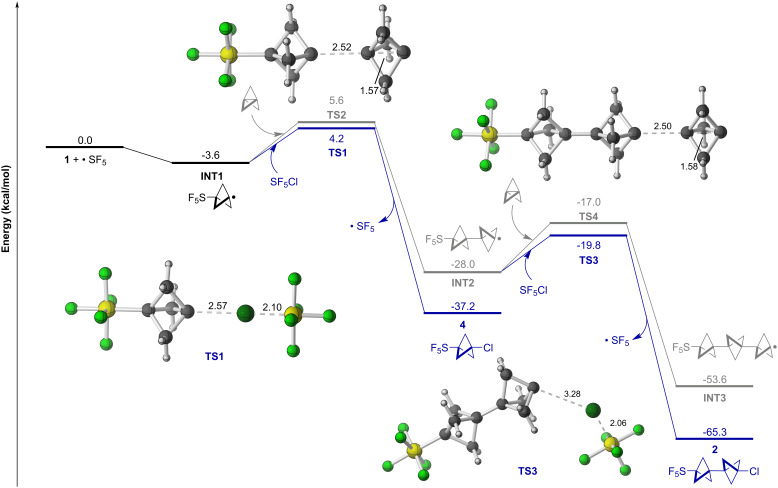
Computed free energy profile for the oligomerization of [1.1.1]propellane (**1**) following SF_5_ radical addition at PWPB95-D4/def2-QZVPP//PCM(Et_2_O)-*ω*B97X-D/def2-TZVP level of theory.

The origin of this innately controlled oligomerization was then investigated through density functional theory (DFT) calculations. The free energy profile of the radical chain propagation sequence was computed at the PWPB95-D4/def2-QZVPP//PCM(Et_2_O)-*ω*B97X-D/def2-TZVP level of theory [51–58] (Figure 2). Following addition of an SF_5_ radical to **1** to form **INT1**, a Cl atom could be abstracted from SF_5_Cl via **TS1** to form **4** or, alternatively, **INT1** could be added to another equiv of **1** via **TS2** to form **INT2**. Although formation of **4** is notably more thermodynamically favorable than **INT2** (ΔΔ*G* = −9.2 kcal/mol), a small difference in activation free energy is predicted (ΔΔ*G*^‡^ = −1.4 kcal/mol). This, at least in part, provides an explanation as to how favoring the path to **INT2** may be achieved in practice through increasing concentration of **1**.

Subsequently, **INT2** could abstract a Cl atom from SF_5_Cl via **TS3** to form **2** or add to a third equiv of **1** through **TS4**, leading to **INT3**. Once again, Cl atom abstraction is thermodynamically (ΔΔ*G* = −11.7 kcal/mol) and kinetically (ΔΔ*G*^‡^ = −2.8 kcal/mol) favored. However, the ΔΔ*G*^‡^ is notably greater in the second product-determining step than the first product-determining step, which is consistent with our experimental observation that **2** forms preferentially over further oligomerization.

For another point of comparison, we examined the reactivity of **1** with CF_3_SF_4_Cl. This reagent is known to behave comparably to SF_5_Cl in radical chain reactions [17,59–61] and can also be prepared conveniently in house [62]. In an analogous equivalents screen, we found that the **5**:**3** product ratio shifts from 7.7:1 using 1.0 equiv of **1** to 1:2.1 using 20 equiv of **1** (Table 2). In the latter scenario, 96% of the material balance could be accounted for in the formation of **5** and **3**, indicating oligomerization is likewise stunted beyond incorporation of two BCP units. Also similar to pentafluorosulfanylation conditions, we found that adding CF_3_SF_4_Cl portion-wise to 6.0 equiv of **1** enables access to **3** in 60% yield by ^19^F NMR (53% isolated). Interestingly, we observed that aryl-SF_4_Cl compounds do not follow the same selectivity trend as SF_5_Cl and CF_3_SF_4_Cl additions, suggesting that the controlled oligomerization phenomenon is quite sensitive to changes in the fluorinated sulfur reagent scaffold (see Supporting Information File 1 for more details).

**Table 2:** Effect of [1.1.1]propellane (**1**) equivalents relative to CF_3_SF_4_Cl on selectivity.^a^

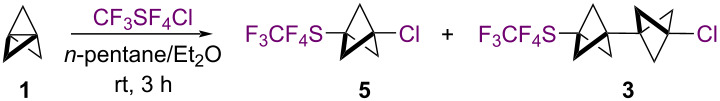				

entry	**1** (equiv)	**5** (% yield)^b^	**3** (% yield)^b^	**5**:**3**

1	1.0	80%	10%	7.7:1.0
2	2.0	71%	21%	3.4:1.0
3	3.0	65%	31%	2.1:1.0
4	4.0	55%	40%	1.4:1.0
5	6.0	49%	40%	1.2:1.0
6	8.0	39%	46%	1.0:1.2
7	10	33%	62%	1.0:1.9
8	20	31%	65%	1.0:2.1
**9** **^c^**	**6.0**	**35%**	**60% (53%)** **^d^**	**1.0:1.7**

^a^A 0.1 M solution of CF_3_SF_4_Cl in *n*-pentane (0.03 mmol) was added to a 0.8 M solution of [1.1.1]propellane in Et_2_O under Ar atmosphere and stirred at rt for 3 h. ^b^Yield determined by ^19^F NMR. ^c^CF_3_SF_4_Cl was added portion-wise. ^d^Isolated yield.

**Figure 3:**
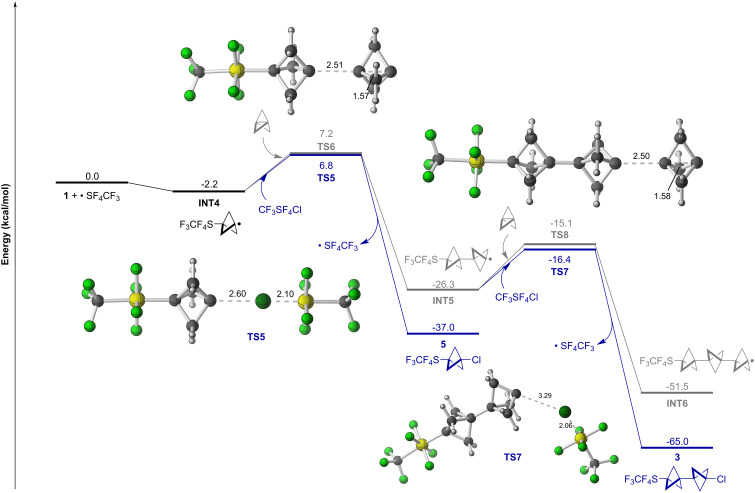
Computed free energy profile for the oligomerization of [1.1.1]propellane (**1**) following CF_3_SF_4_ radical addition at the PWPB95-D4/def2-QZVPP//PCM(Et_2_O)-*ω*B97X-D/def2-TZVP level of theory.

This second instance of controlled oligomerization of **1** using CF_3_SF_4_Cl was also studied at the PWPB95-D4/def2-QZVPP//PCM(Et_2_O)-*ω*B97X-D/def2-TZVP level of theory (Figure 3). Addition of a CF_3_SF_4_ radical to **1** affords **INT4**, which can either abstract a Cl atom from CF_3_SF_4_Cl via **TS5** to make **5** or add to another equiv of **1** via **TS6** to access **INT5**. As anticipated, chlorination of the radical is thermodynamically favored over addition to **1** (ΔΔ*G* = −10.7 kcal/mol). It is also predicted here that the free energy of activation is lower for chlorination, albeit only by 0.4 kcal/mol. This is consistent with the notion that the kinetic preference can be overcome by increasing the concentration of **1** relative to CF_3_SF_4_Cl. In the second product-determining step, Cl atom abstraction by **INT5** via **TS7** to make **3** is kinetically favorable over addition of a third equiv of **1** via **TS8** to access **INT6**, although the preference is not as large as for formation of **2** (ΔΔ*G*^‡^ = −1.3 kcal/mol).

Thus, the predicted trend for both SF_5_Cl and CF_3_SF_4_Cl additions across **1** indicates a stronger preference for Cl atom abstraction over continued oligomerization in the second product-determining step than in the first – in line with our experimental observations. One possible explanation for this phenomenon is rooted in better radical polarity matching after incorporation of the second BCP unit [37–38]. That is, the carbon-centered radicals in both **INT1** and **INT4** are closer to strong electron-withdrawing groups than are the radical centers in **INT2** and **INT5**, rendering **INT1** and **INT4** relatively more electrophilic. Inductive effects drop off steeply with distance, and it is also established that a substituent (or, e.g., a radical or cation) on the transannular carbon atom of a bicyclopentyl moiety can interact through space [35,63–64]. The consequence is ostensibly that more "nucleophilic" **INT2** and **INT5** are better matched for Cl atom abstraction from the "electrophilic" reagent (SF_5_Cl or CF_3_SF_4_Cl).

To test this hypothesis, we examined computed trends in various electronic parameters for the **INT1**–**INT3** and the **INT4**–**INT6** series (Table 3). For instance, across several charge models (i.e., Hirshfeld [65], NPA [66–68], and CHELPG [69]), the charge (q) on the carbon atom on which the radical is centered becomes more negative (or less positive) the farther it is from either the SF_5_ or CF_3_SF_4_ substituent, consistent with the notion that it becomes more nucleophilic. Moreover, the Δq is largest between the first two intermediates in both series – **INT1** vs **INT2** and **INT3** vs **INT4** – indicating that the most dramatic change in bicyclopentyl radical philicity would arise after incorporation of the second BCP unit.

**Table 3:** Key indices computed to compare reactivity. Partial charges (q) and condensed Fukui functions are evaluated at the reacting carbon or chlorine atom and are in units of elementary charge (*e*).^a^

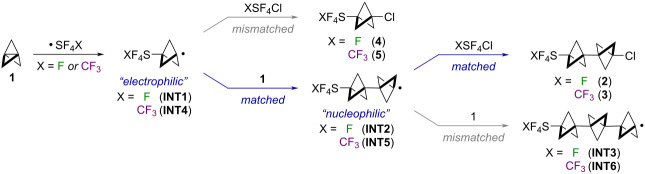						

compound	q(Hirshfled) (e)	q(NPA) (e)	q(CHELPG) (e)	f ^0^ (e)^b^	ω (*eV*)^c^	N (*eV*)^d^

**1**	−0.090	−0.069	−0.255	0.235	0.82	1.70
SF_5_Cl	−0.055	−0.160	0.030	0.534	2.48	−0.74
CF_3_SF_4_Cl	−0.061	−0.150	0.056	0.527	2.52	−0.66
**INT1**	−0.019	0.092	−0.125	0.307	2.70	2.95
**INT2**	−0.053	0.073	−0.196	0.347	1.78	3.58
**INT3**	−0.060	0.065	−0.214	0.345	1.66	3.77
**INT4**	−0.019	0.092	−0.144	0.301	2.74	2.97
**INT5**	−0.053	0.073	−0.193	0.346	1.78	3.59
**INT6**	−0.060	0.064	−0.214	0.345	1.65	3.77

^a^Calculations performed at the PCM(Et_2_O)-ωB97X-D/def2-TZVP level of theory. ^b^Radical Fukui function. ^c^Electrophilicity index. ^d^Nucleophilicity index.

In addition to charge models, we evaluated global reactivity indices (*ω*: electrophilicity index [70] and N: nucleophilicity index [71]) within the conceptual density functional theory (CDFT) framework [72–74]. The data show that BCP has a higher N value – thus stronger nucleophilic tendency – compared to both SF_5_Cl and CF_3_SF_4_Cl. Conversely, comparison of *ω* values shows significantly higher electrophilicity of SF_5_Cl and CF_3_SF_4_Cl compared to BCP. These results, coupled with decreasing *ω* and increasing N when more BCP units are incorporated, lend qualitative support to our radical polarity matching hypothesis. Moreover, assessment of radical Fukui functions (*f*^0^) [75] indicates that both SF_5_Cl and CF_3_SF_4_Cl are intrinsically more susceptible to radical attack than **1**, which is consistent with the lower computed barriers for Cl atom abstraction in each case.

These computed trends also potentially account for the fact that selectivity for the [2]staffane (i.e., truncated oligomerization) using aryl-SF_4_Cl reagents was not observed. On the basis of CDFT results, a model aryl-SF_4_Cl compound (i.e., 5-chloropyrimidyl-SF_4_Cl) was determined to be significantly less "electrophilic" than SF_5_Cl or CF_3_SF_4_Cl, consistent with a reduction in the radical polarity matching effect (see Supporting Information File 1 for details). This was difficult to predict or rationalize based on calculation of free energies of activation alone. Overall, our results suggest that this alternating polarity matching effect is subtle and subject to mitigation yet can lead to desirable products if employed thoughtfully.

Lastly, following our synthetic and computational studies, accessing a CF_3_SF_4_-containing [2]staffane in good yield and for the first time created an opportunity for structural analysis. We previously reported and contextualized single-crystal X-ray diffraction (SC-XRD) data on **2** [36]; thus, we proceeded to grow crystals of **3** suitable for X-ray analysis through slow evaporation of ethyl acetate.

**Figure 4:**
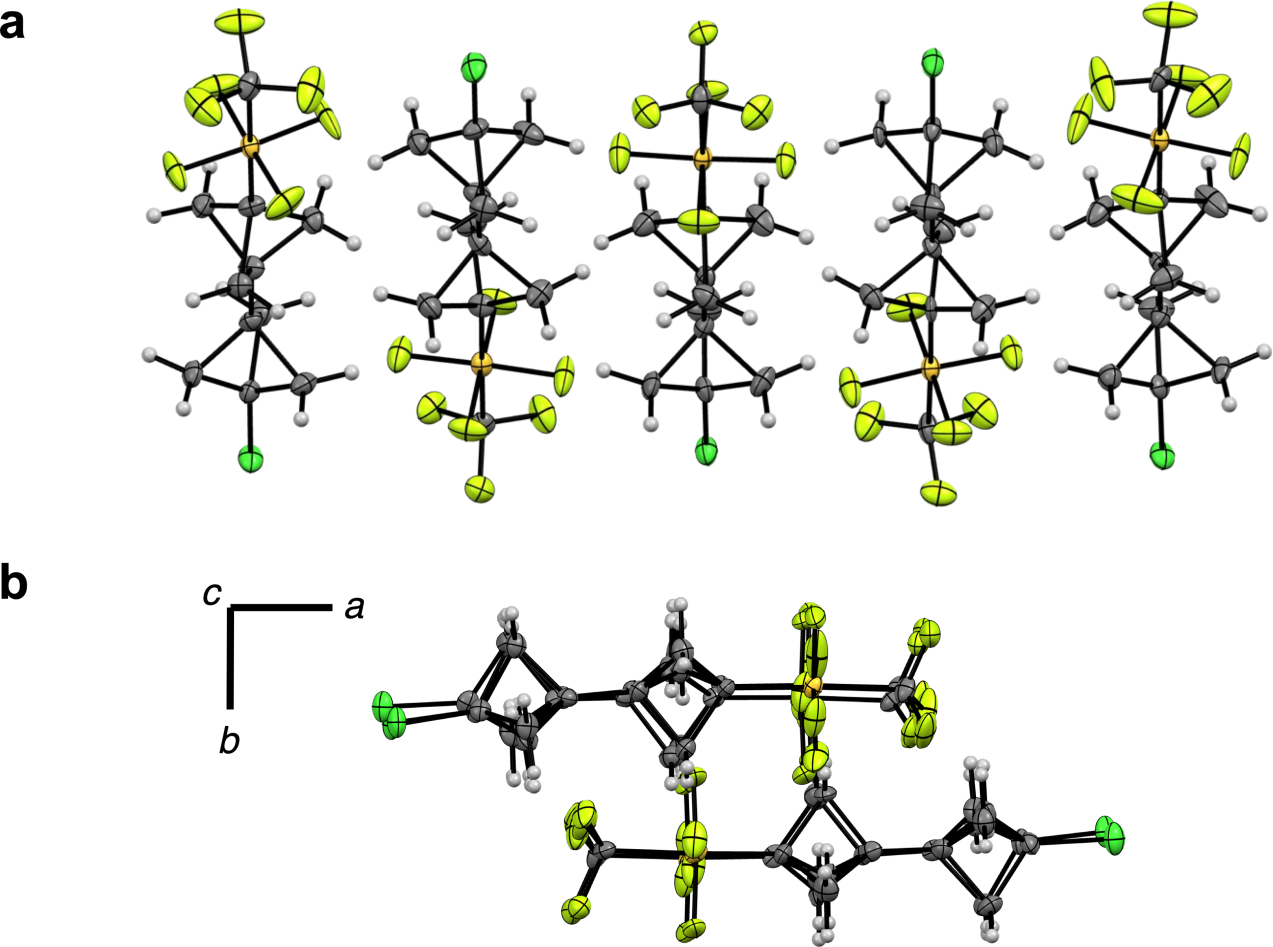
(A) The molecular structure of **3** at 90 K with 5 independent moieties in the asymmetric axis viewed along the *b*-axis. (B) The asymmetric unit of **3** at 90 K viewed along the *c*-axis. Thermal displacement ellipsoids depicted at 50% probability.

To our surprise, an initial measurement of **3** at 90 K revealed an unusually large unit cell (*a* = 7.14 Å, *b* = 21.38 Å, and *c* = 44.04 Å). Following structure solution and refinement [76], we found that **3** crystallizes in an orthorhombic space group *P*2_1_2_1_2_1_ with five symmetry independent moieties (*Z*' = 5) and with no solvent present in the unit cell as an inversion twin (Figure 4). After close examination of the model, we noticed that the *c*-axis was roughly divisible by five with a substructure of *Z*' = 1. This suggested that the *Z*' = 5 unit cell may be due to a phase transition caused by anisotropic contraction [77].

In fact, we confirmed that a phase transition had occurred following structure determination at 240 K [78–79]. The X-ray data revealed that **3** crystallizes in the centrosymmetric orthorhombic space group *Pnma* in the high temperature phase (HTP) with cell axes *a* = 21.56 Å, *b* = 8.95 Å, and *c* = 7.28 Å, in contrast to *P*2_1_2_1_2_1_ in the low temperature phase (LTP). Note that the *b*-axis is roughly 1/5 of the *c*-axis observed at 90 K (the axial rearrangement is due to the change in space group). To discern the approximate temperature of the phase transition, the unit cell was measured in 20 K increments upon cooling from 260 K down to 100 K; additional details are reported in Supporting Information File 1. Interestingly, the original LTP unit cell was not detected; instead, only the reduced cell observed in the HTP was found at all temperatures. However, after warming the same crystal of **3** back to room temperature, it was rapidly cooled to 100 K under a stream of N_2_ and the larger, disordered cell was observed once more [80]. (These observations also prompted us to measure a structure of **2** at 240 K; in this case, the unit cell is virtually identical at both high and low temperatures, indicating no phase transition had occurred – see Supporting Information File 1 for details.)

Accordingly, we gather that the rate of cooling plays an important role whereby rapid cooling effectively "shocks" the crystal of **3**, compressing the unit cell isotropically, and ultimately leads to more disorder in the asymmetric unit [81]. This unexpected observation suggests that CF_3_SF_4_-containing [2]staffanes, in particular, warrant additional studies and may be of interest, e.g., in liquid crystal design.

## Conclusion

Suppressing [*n*]staffane formation beyond *n* = 1 in radical chain reactions involving [1.1.1]propellane (**1**) tends to be more manageable than controlling oligomerization. However, under the right circumstances, alternating radical polarity matching throughout the chain propagation steps could be one way to theoretically "switch off" oligomerization beyond formation of a [2]staffane. Using this logic, our synthetic and computational study demonstrates that selective one-pot syntheses of [2]staffanes can be achieved when employing reagents that serve as radical sources of "extreme" electron-withdrawing groups (e.g., SF_5_ or CF_3_SF_4_), which impact relative philicities of the bicyclopentyl radical intermediates. Over the course of this study, we also found that the SF_5_- and CF_3_SF_4_-containing [2]staffanes reported herein are structurally interesting in their own right. Future work will examine potential applications of **2** and **3** and explore tactics for C–Cl bond functionalization.

## Supporting Information

File 1 Experimental procedures, characterization data, NMR spectra, computational details, and X-ray crystallographic experimental details.

File 2 LTP X-ray crystal structure of compound **3** (2357079.cif), HTP X-ray crystal structure of compound **3** (2357080.cif) and X-ray crystal structure of compound **2** at 240 K (238115.cif).

## Data Availability

All experimental data that supports the findings of this study are available in the published article and/or the supporting information to this article; coordinates for computed structures are openly available in ioChem-BD at https://doi.org/10.19061/iochem-bd-6-384.
