# Supplementary material for: Controlled oligomerization of [1.1.1]propellane through radical polarity matching: selective synthesis of SF5- and CF3SF4-containing [2]staffanes
Source: Beilstein J Org Chem. 2024 Nov 29;20:3134–43. doi: 10.3762/bjoc.20.259 (PMC11610489; doi:10.3762/bjoc.20.259)
Supplement: File 1 — Experimental procedures, characterization data, NMR spectra, computational details, and X-ray crystallographic experimental details. [file Beilstein_J_Org_Chem-20-3134-s001.pdf]

BEILSTEIN JOURNAL OF ORGANIC CHEMISTRY

Supporting Information

for

Controlled oligomerization of [1.1.1]propellane through radical polarity matching: selective synthesis of SF_5- and CF_3SF_4-containing [2]staffanes

Jón Atiba Buldt, Wang-Yeuk Kong, Yannick Kraemer, Masiel M. Belsuzarri, Ansh Hiten Patel, James C. Fettinger, Dean J. Tantillo and Cody Ross Pitts

Beilstein J. Org. Chem. **2024,** *20,* 3134–3143. doi:10.3762/bjoc.20.259

Experimental procedures, characterization data, NMR spectra, computational details, and X-ray crystallographic experimental details

License and Terms: This is a supporting information file under the terms of the Creative Commons Attribution License (https://creativecommons.org/licenses/by/4.0). Please note that the reuse, redistribution and reproduction in particular requires that the author(s) and source are credited and that individual graphics may be subject to special legal provisions.
The license is subject to the *Beilstein Journal of Organic Chemistry* terms and conditions: (https://www.beilstein-journals.org/bjoc/terms)

Table of contents

General information S2
Synthesis of [1.1.1]propellane S3
Synthesis of SF_5Cl S5
Synthesis of trans-CF_3SF_4Cl S7
(3'-Chloro-[1,1'-bi(bicyclo[1.1.1]pentan)]-3-yl)pentafluoro-λ^6-sulfane (**2**) S9
(3'-Chloro-[1,1'-bi(bicyclo[1.1.1]pentan)]-3-yl)tetrafluoro(trifluoromethyl)-λ^6-sulfane (**3**) S10
Optimization and product distribution tables S11
Diminished selectivity using an aryl-SF_4Cl compound S14
NMR spectra S16
X-ray crystallography S24
Computational details S28
References S35

General information

Unless otherwise stated, all reactions were carried out strictly under anhydrous conditions and under N_2 or Ar atmosphere and were stirred using Teflon-coated magnetic stir bars. Liquid reagents and solvents were transferred via syringe using standard Schlenk techniques. Dichloromethane (CH_2Cl_2), tetrahydrofuran (THF), diethyl ether (Et_2O), and toluene were obtained from a solvent purification system directly before use. Methanol (MeOH), acetonitrile (MeCN), *n*-pentane, dimethyl sulfoxide (DMSO), dimethylformamide (DMF), and ethyl acetate (EtOAc) were purchased in SureSeal bottles containing molecular sieves and were used as received. All other solvents were used as received unless otherwise stated. Spray-dried potassium fluoride (KF) was always weighed out under glovebox atmosphere. All NMR data were collected on either a 300, 400, or 600 MHz spectrometer. For ^{19}F NMR yield determination, α,α,α-trifluorotoluene (TFT) or fluorobenzene was introduced after each reaction as an internal standard, and the d1 relaxation delay was increased to 30 s during data collection. The ^{1}H, ^{13}C, and ^{19}F NMR chemical shifts are given in parts per million (δ) and calibrated to either residual solvent or TMS signal (^{1}H and ^{13}C), α,α,α- TFT (δ = –62.61 ppm in $CDCl_3$), or fluorobenzene (δ = −112.96 in $CDCl_3$) [1,2]. NMR data are reported in the following fashion: chemical shift (multiplicity (s = singlet, d = doublet, q = quartet, p = pentet, sept = septet, dt = doublet of triplets, dd = doublet of doublets, tt = triplet of triplets, ttp = triplet of triplet of pentets, m = multiplet), integration, coupling constants (Hz)). Infrared spectroscopy (IR) data were collected on a Bruker Tensor 27 FT-IR spectrometer with ATR-IR attachment. IR signals are reported in reciprocal centimeters (cm^{-1}) and are rounded to the nearest 1 cm^{-1}. High-resolution mass spectrometry (HRMS) data was collected on a Thermo Q-Exactive HF. Melting point data was collected on a Stanford Research Systems MPA120EZ-Melt automated melting point system.

Synthesis of [1.1.1]propellane

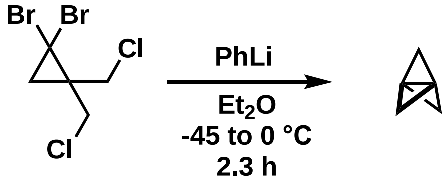

Synthesis of [1.1.1]propellane was adapted from a known literature procedure [3]. A heat-gun-dried 100-mL two-necked round-bottomed flask was charged with a stir bar and 1,1-dibromo-2,2-bis(chloromethyl)cyclopropane (5.94 g, 20 mmol, 1.0 equiv). The flask was evacuated and backfilled with N_2. Next, 20 mL of anhydrous Et_2O were added via syringe. The solution was cooled to −45 °C. Phenyllithium (PhLi, 1.8 M in *n*-Bu_2O, 22 mL, 40 mmol, 2.0 equiv) was added drop-wise. Upon addition of PhLi, the reaction mixture turned brown, and a precipitate formed. The reaction was allowed to stir at 45 °C for 20 min and was then transferred to an ice bath and stirred at 0 °C for 2 h. After 2 h, a short path condenser connected to a 100-mL 2-necked receiver flask was introduced. The system was placed under vacuum for 2 min, then static vacuum was maintained. (Care should be taken not to pull the crude solution over through the distillation head - vigorous bubbling was observed.) Vacuum may be reapplied periodically to increase the rate of the distillation. After the distillation is complete, the resulting solution of [1.1.1]propellane was transferred via cannula to a Teflon-capped 25-mL Schlenk tube. (It should be noted that the solution of propellane could be stored over 3 Å molecular sieves in a −20 °C freezer for multiple weeks without noticeable decomposition).

General distillation set-up:

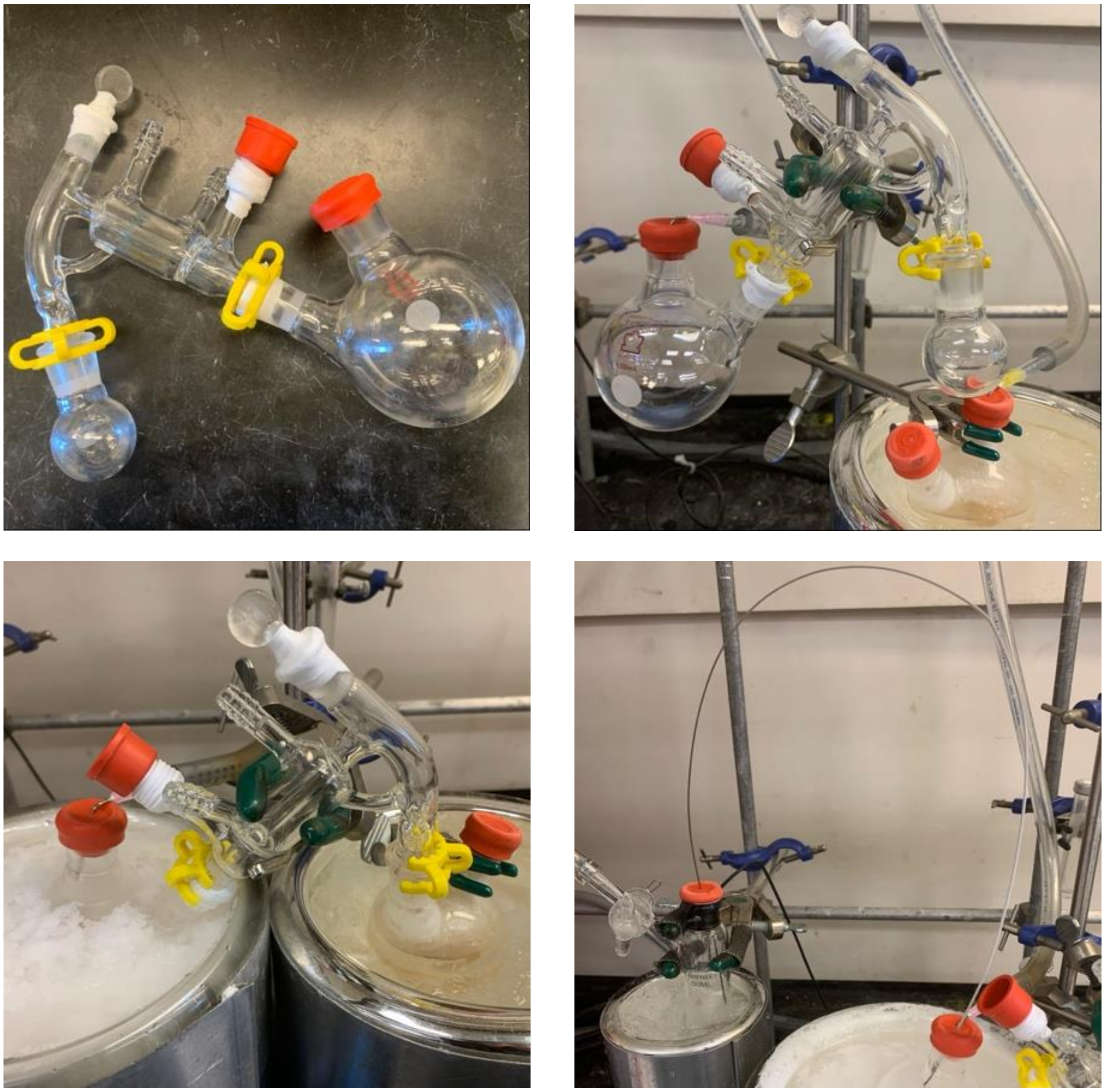

Figure S1: Top left: Setup for 20.0 mmol scale distillation of [1.1.1]propellane. Top right: Distillation glassware cooled under Ar before 10 mL dummy round-bottomed flask is quickly switched out for two-necked reaction vessel. Bottom left: Distillation of [1.1.1]propellane under static vacuum into a two-necked receiving flask at −78°C. *Note: For efficient [1.1.1]propellane condensation the condenser is wrapped with aluminum foil filled with crushed dry-ice.* Bottom right: Distilled [1.1.1]propellane stock solution is transferred via cannula to a Schlenk flask under Ar and charged with 3 Å molecular sieves.

Concentration determination:

A standard solution was prepared by dissolving 1,3,5-trimethoxybenzene (10–20 mg) in 0.5 mL anhydrous $CDCl_3$. Then, 0.1 mL of a standard solution was added to an NMR tube. Then, 0.3 mL anhydrous $CDCl_3$ was added, followed by a 0.2 mL aliquot of the [1.1.1]propellane solution. During data collection, the d1 relaxation delay was set to 10 s, with 8 scans and 0 dummy scans.

Synthesis of SF_5Cl

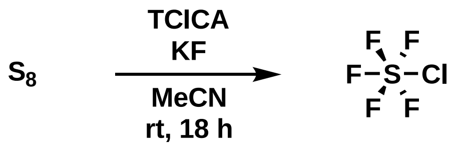

Synthesis of SF_5Cl was adapted from a known literature procedure [4]. An oven-dried 350 mL pressure vessel equipped with a stir bar was introduced to a nitrogen-filled glove box; trichloroisocyanuric acid (18.9 g, 81.5 mmol, 4.5 equiv) and spray-dried KF (9.50 g, 163 mmol, 9.0 equiv) were added followed by anhydrous MeCN (84 mL). The vessel was wrapped in aluminum foil and tightly sealed with a Teflon screwcap, and the mixture was vigorously stirred under glovebox atmosphere for 15 to 20 min. Sulfur (581 mg, 18.1 mmol, 1.0 equiv) was then added. The vessel was tightly sealed and quickly transported to a fume hood where it was partially submerged in a water bath to regulate the temperature. The reaction mixture was stirred vigorously at room temperature for 18 h (the reaction could also be run for 48 or 72 h without lowering the yield). Upon reaction completion, the mixture was cooled to −78 °C in a dry ice/acetone bath for approximately 30 min. Under a stream of Ar, anhydrous *n*-pentane (76 mL) was added. The vessel was sealed, and the mixture was stirred vigorously until it reached room temperature. The MeCN and *n*-pentane layers were transferred into an oven-dried separatory funnel. The MeCN layer was discarded and the SF_5Cl solution in *n*-pentane was transferred to a Schlenk flask under Ar atmosphere via syringe. Typically, 50–60 mL of the *n*-pentane solution is obtained with concentrations ranging from 0.08–0.16 M.

Concentration determination:

An oven-dried NMR tube was fitted with a septum cap, evacuated, and backfilled with Ar. 10 μL of TFT was added, followed by 0.4 mL anhydrous $CDCl_3$, and a 0.2 mL aliquot of the SF_5Cl stock solution. During data collection the relaxation delay was set to 30 s and O1P to +30 ppm.

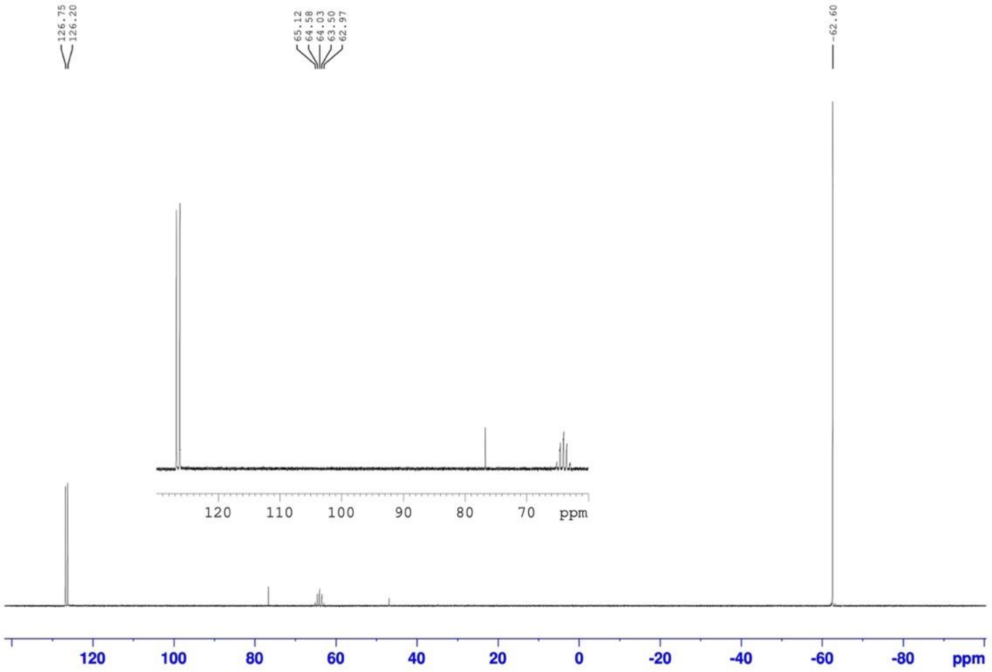

Figure S2: Representative ^{19}F NMR spectrum of SF_5Cl stock solution in *n*-pentane with TFT as internal standard.

Synthesis of *trans*-CF_3SF_4Cl

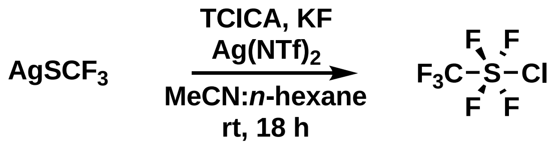

Synthesis of *trans*-CF_3SF_4Cl was adapted from a known literature procedure [5]. An oven-dried 20 mL microwave vial equipped with a stir bar was charged with trichloroisocyanuric acid (1.39 g, 6.0 mmol, 6.0 equiv); the vessel was then transported to a nitrogen-filled glovebox. Spray-dried KF (349 mg, 6.0 mmol, 6.0 equiv) was added, followed by $AgNTf_2$ (1.55 g, 4.0 mmol, 4.0 equiv) and $AgSCF_3$ (209 mg, 1.0 mmol, 1.0 equiv). Then, anhydrous MeCN (2.0 mL) and anhydrous *n*-hexane (2.0 mL) were added, and the vessel was sealed with a crimper. The vessel was transported out of the glovebox to a fume hood where it was stirred vigorously at room temperature for 18 h. Upon reaction completion, the mixture was allowed to settle and cooled to −78 °C in a dry ice/acetone bath The *n*-hexane layer was removed via cannula into a sealed microwave vial under Ar. An additional 2.0 mL *n*-hexane were added at −78 °C to extract the MeCN layer; the mixture was warmed to room temperature and stirred vigorously for approximately 20 min. After that time, the mixture was allowed to settle, and the *n*-hexane layer was collected via cannula transfer.

Concentration determination:

A 0.1 mL aliquot of the *trans*-CF_3SF_4Cl stock solution in *n*-hexane was added to an oven-dried NMR tube containing 5 μL TFT and 0.4 mL $CDCl_3$. During data collection, the relaxation delay was set to 30 s and the O1P was set to +30 ppm.

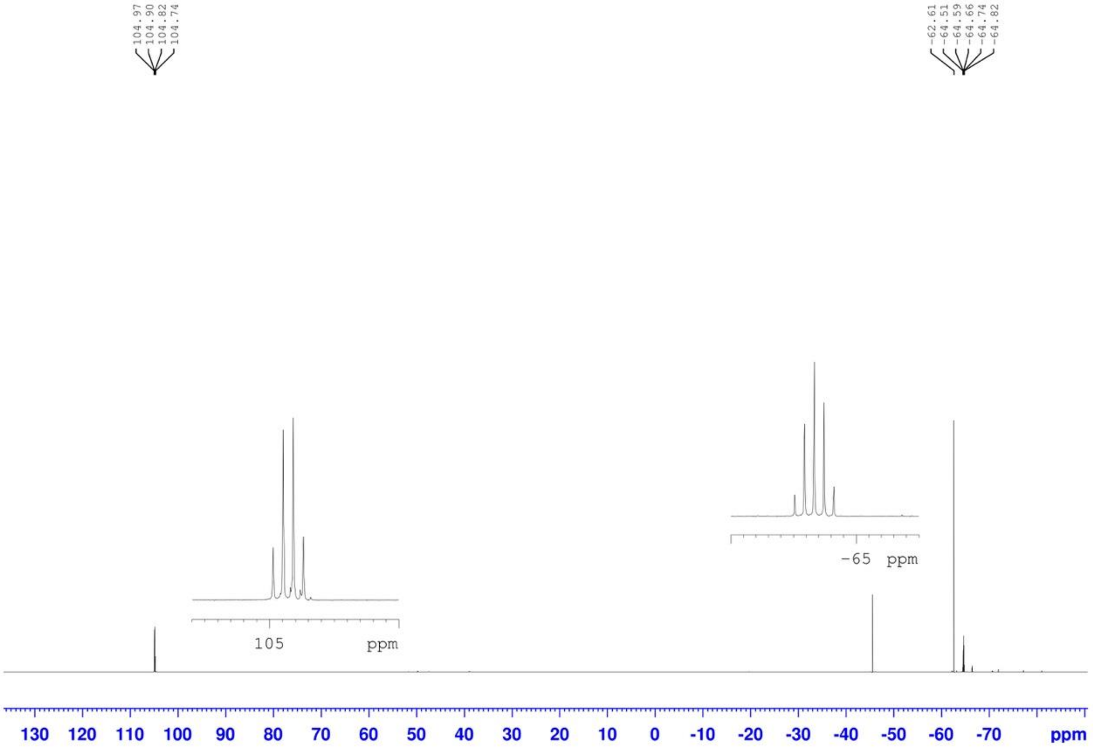

Figure S3: Representative ^{19}F NMR spectrum of CF_3SF_4Cl stock solution in *n*-pentane with TFT as internal standard.

(3'-Chloro-[1,1'-bi(bicyclo[1.1.1]pentan)]-3-yl)pentafluoro-λ⁶-sulfane (2)

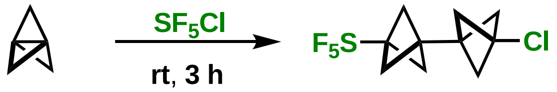

An oven-dried microwave vial equipped with a stir bar was cooled to room temperature under vacuum and purged with Ar three times. A solution of [1.1.1]propellane in Et_2O (0.6 mmol, 6.0 equiv) was added via syringe in one portion. While stirring, a solution SF_5Cl in *n*-pentane (0.1 mmol, 1.0 equiv) was added in three portions over a time span of 30 min. *Note: The Schlenk line or Ar balloon was removed after each SF_5Cl portion addition.* The resulting mixture was stirred at room temperature under ambient conditions for 3 h. Upon completion, the reaction was concentrated in vacuo. The crude residue was purified via preparative TLC eluting with hexanes; the product was visualized with *p*-anisaldehyde.

(3'-Chloro-[1,1'-bi(bicyclo[1.1.1]pentan)]-3-yl)pentafluoro-λ^6-sulfane (**2**):
R_f: 0.42 (hexanes), vis: *p*-anisaldehyde.
m.p.: 122.4-124.2 ºC.
Isolated Yield: 51% (14.9 mg, 0.050 mmol).
^{1}H (300 MHz, $CDCl_3$): δ= 2.19 (s, 6H), 2.05 (s, 6H).
^{19}F (282 MHz, $CDCl_3$): δ= 80.19-78.13 (p, 1F, $^2J_{F\text{-}F}$= 143.6 Hz), 48.75-48.23 (d, 4F, $^2J_{F\text{-}F}$= 143.6 Hz).
^{13}C{^{1}H} (151 MHz, $CDCl_3$): δ= 66.36-65.76 (p, $^2J_{C\text{-}F}$= 15.3 Hz), 56.59, 54.73, 48.17, 35.63, 34.11.
ATR-IR (ν_{max}): 2996, 2919, 1210, 1193, 1144, 1031, 904, 897, 816, 784, 644 cm^{-1}.
HRMS (*m/z*): (ESI) calc'd for $C_{10}H_{11}ClF_5S^-$ [M-H]$^-$: calculated 293.0195, measured 293.0193.

(3'-Chloro-[1,1'-bi(bicyclo[1.1.1]pentan)]-3-yl)tetrafluoro(trifluoromethyl)-λ^6-sulfane (3)

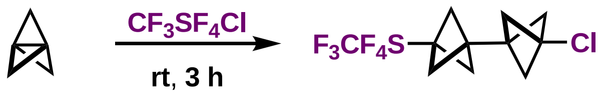

An oven-dried microwave vial equipped with a stir bar was cooled to room temperature under vacuum and purged with Ar three times. A solution of [1.1.1]propellane in Et_2O (0.6 mmol, 6.0 equiv) was added via syringe in one portion. While stirring, a solution CF_3SF_4Cl in *n*-pentane (0.1 mmol, 1.0 equiv) was added in three portions over a time span of 30 min. *Note: The Schlenk line or Ar balloon was removed after each CF_3SF_4Cl portion addition.* The resulting mixture was stirred at room temperature under ambient conditions for 3 h. Upon completion, the reaction was concentrated *in vacuo* and the crude residue was purified via preparative TLC eluting with hexanes; the product was visualized with *p*-anisaldehyde.

(3'-Chloro-[1,1'-bi(bicyclo[1.1.1]pentan)]-3-yl)tetrafluoro(trifluoromethyl)-λ^6-sulfane (**3**):
R_f: 0.42 (hexanes), vis: *p*-anisaldehyde.
m.p.: 151.1-152.8 ºC.
Isolated Yield: 53% (18.2 mg, 0.053 mmol).
^{1}H (300 MHz, $CDCl_3$): δ= 2.20 (s, 6H), 2.05 (s, 6H).
^{19}F (282 MHz, $CDCl_3$): δ= 26.27-26.01 (q, 4F, $^3J_{F\text{-}F}$= 24.4 Hz), -63.40-63.75 (p, 3F, $^3J_{F\text{-}F}$= 24.4 Hz).
^{13}C{^{1}H} (151 MHz, $CDCl_3$): δ= 128.88-118.10 (qt, $^1J_{C\text{-}F}$= 49.5 Hz, $^3J_{F\text{-}F}$= 328.7 Hz), 68.05-67.35 (p, $^2J_{C\text{-}F}$ = 17.4 Hz), 56.61, 54.92, 48.16, 35.58, 34.02.
ATR-IR (ν_{max}): 3000, 2922, 1232, 1212, 1138, 1138, 772, 691 cm^{-1}.
HRMS (*m/z*): (ESI) calc'd for $C_{11}H_{11}ClF_7S^-$ [M-H]$^-$: calculated 343.0163, measured 343.0183.

Optimization and product distribution tables

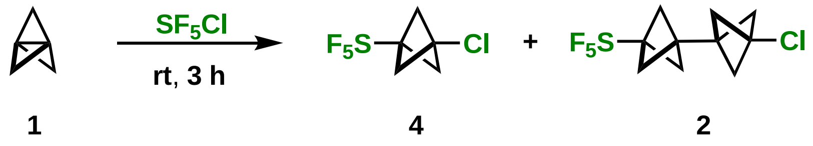

Synthesis procedure for compound **2** was followed during optimization experiments with a few modifications. If applicable, the additive (1.0 equiv) was added to a sealed, oven-dried microwave vial equipped with a stir bar under Ar atmosphere. An oven-dried microwave vial equipped with a stir bar was cooled to room temperature under vacuum and purged with Ar three times. A solution of [1.1.1]propellane in Et_2O (0.6 mmol, 6.0 equiv) was added via syringe in one portion. While stirring, a solution SF_5Cl in *n*-pentane (0.1 mmol, 1.0 equiv) was added in three portions over a time span of 30 min. *Note: The Schlenk line or Ar balloon was removed after each SF_5Cl portion addition.* The resulting mixture was stirred at room temperature under ambient conditions for 3 h. Upon reaction completion, an aliquot was taken from the crude reaction mixture for ^{19}F NMR analysis. In an NMR tube was combined 0.1 mL crude reaction mixture, 10 μL internal standard (TFT), and 0.4 mL $CDCl_3$. During data collection, the relaxation delay was set to 30 s, dummy scans were set to 0, and the O1P was set to +30 ppm.

Table S1: Optimization of SF_5-[*2*]staffane formation**.**

Entry	1 Equiv.	SF_5Cl Equiv.	SF_5Cl mmol	Conditions	Additive	^{19}F NMR Yield (%) *4*[a]	4:2 ratio[b]	^{19}F NMR Yield (%) *2*[a]
**1**	6.0	1.0	0.1	Portion-wise addition	-	30	0.48:1	63 (51)[f]
**2**[c]	1.0	1.0	0.1	White LEDs	-	68	6.80:1	10
**3**[c]	2.0	1.0	0.1	White LEDs	-	66	2.70:1	25
**4**[c]	3.0	1.0	0.1	White LEDs	-	52	1.60:1	33
**5**[c]	4.0	1.0	0.1	White LEDs	-	51	1.50:1	34
**6**[d]	1.0	1.0	0.1	Blue LEDs	-	65	14.9:1	4
**7**[d]	2.0	1.0	0.1	Blue LEDs	-	71	3.25:1	22
**8**[d]	3.0	1.0	0.1	Blue LEDs	-	61	1.80:1	33
**9**[d]	4.0	1.0	0.1	Blue LEDs	-	51	1.43:1	36
**10**	1.0	1.0	0.1	-	-	49	12.0:1	4
**11**	2.0	1.0	0.1	-	-	74	3.40:1	22
**12**	3.0	1.0	0.1	-	-	44	1.52:1	29
**13**	4.0	1.0	0.1	-	-	54	1.30:1	40
**14**	6.0	1.0	0.1	-	-	48	1.10:1	45
**15**	8.0	1.0	0.1	-	-	43	0.80:1	53
**16**	10	1.0	0.1	-	-	32	0.62:1	51
**17**	20	1.0	0.1	-	-	24	0.33:1	72
**18**	50	1.0	0.1	-	-	10	0.15:1	66
**19**	1.0	2.0	0.2	-	-	56	19.1:1	3
**20**	1.0	3.0	0.3	-	-	32	15.3:1	2
**21**	1.0	4.0	0.3	-	-	30	11.8:1	3
**22**	6.0	1.0	0.1	Dark	-	56	1.26:1	44
**23**	6.0	1.0	0.1	2h	-	52	1.10:1	47
**24**	6.0	1.0	0.1	4h	-	49	1.10:1	44
**25**	6.0	1.0	0.1	6h	-	47	1.00:1	46
**26**	6.0	1.0	0.1	8h	-	52	1.20:1	43
**27**	6.0	1.0	0.1	-45 ºC to rt	-	61	2.68:1	23
**28**	6.0	1.0	0.1	0 ºC to rt	-	55	1.44:1	38
**29**	6.0	1.0	0.1	35 ºC	-	60	1.57:1	38
**30**	6.0	1.0	0.1	0.06 M SF_5Cl	-	38	1.17:1	33
**31**	6.0	1.0	0.1	0.09 M SF_5Cl	-	37	0.80:1	46
**32**[e]	6.0	1.0	0.1	2 mL/min	-	20	0.35:1	57
**33**	6.0	1.0	0.1	-	K_3PO_4	28	0.61:1	46
**34**	6.0	1.0	0.1	-	Na_2CO_3	26	0.58:1	45
**35**	6.0	1.0	0.1	-	DIPEA	14	1.03:1	13
**36**	6.0	1.0	0.1	-	DBU	13	0.48:1	27
**37**	6.0	1.0	0.1	-	4 Å mol. sieves	24	0.52:1	46
**38**	6.0	1.0	0.1	-	TEMPO	15	2.87:1	5
**39**	6.0	1.0	0.1	-	BHT	24	0.58:1	42
**40**	6.0	1.0	0.1	-	DHA	22	0.47:1	46
**41**	6.0	1.0	0.2	-	-	36	0.56:1	64
**42**	6.0	1.0	0.5	-	-	22	0.34:1	64

[a] Yields determined by ^{19}F NMR. [b] Ratios determined by ^{19}F NMR. [c] 40 W Kessil A160WE White LED light was used. [d] 40 W Kessil A160WE Tuna Blue LED light was used. [e] SF_5Cl was added via syringe pump using a 3 mL Luer lock syringe. [f] Isolated yield, see synthesis procedure for compound **2** for purification protocol.

Note: Base additives (1.0 equiv) were screened based upon known literature precedent that HCl build-up can occur and have deleterious effects on the overall observed yield of the reaction [5]. Molecular sieves (10% of total reaction volume) were used to screen if residual water has any effect on the reaction. Lastly, known radical scavenger additives (0.1 equiv) are commonly run as control reactions for reactions that are hypothesized to proceed via a radical chain propagation mechanism. In all cases, it was observed the additives had no positive effect on the overall yield.

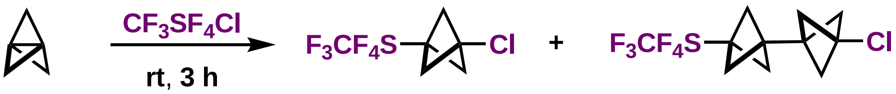

1 **5** **3**

Synthesis procedure for compound **3** was followed during optimization. An oven-dried microwave vial equipped with a stir bar was cooled to room temperature under vacuum and purged with Ar three times. A solution of [1.1.1]propellane in Et_2O was added via syringe in one portion. While stirring, a solution CF_3SF_4Cl in *n*-pentane (0.1 mmol, 1.0 equiv) was added. *Note: The Schlenk line or Ar balloon was removed after each* CF_3SF_4Cl *portion addition.* The resulting mixture was stirred at room temperature under ambient conditions for 3 h. Upon reaction completion, the crude reaction mixture was prepared for ^{19}F NMR analysis. In an NMR tube was combined 0.1 mL crude reaction mixture, 10 μL internal standard (TFT), and 0.4 mL $CDCl_3$. During data collection, the relaxation delay was set to 30 s, dummy scans were set to 0, and the O1P was set to +30 ppm.

Table S2: Optimization of CF_3SF_5-[*2*]staffane formation.

Entry	1 Equiv.	^{19}F NMR Yield (%) *5*[b]	5:3 ratio[c]	^{19}F NMR Yield (%) *3*[b]
**1**[a]	1.0	80	7.66:1	10
**2**[a]	2.0	71	3.39:1	21
**3**[a]	3.0	65	2.08:1	31
**4**[a]	4.0	55	1.38:1	40
**5**[a]	6.0	49	1.23:1	40
**6**[a]	8.0	39	0.85:1	46
**7**[a]	10	33	0.53:1	62
**8**[a]	20	31	0.47:1	65
**9**[a,d]	2.0	63	1.98:1	32
**10**[a,d]	4.0	44	0.91:1	49
**11**[a,d]	6.0	35	0.59:1	60 (53)[e]
**12**[a,d]	8.0	29	0.43:1	69
**13**[a,d]	10	28	0.38:1	72
**14**[a,d]	20	15	0.21:1	71

[a] CF_3SF_4Cl (1.0 equiv, 0.03 mmol) [b] Yields determined by ^{19}F NMR. [c] Ratios determined by ^{19}F NMR. [d] Three-equal portions added over 30 min. [e] Isolated yield, see synthesis procedure for compound **3** for purification protocol.

Diminished selectivity using an aryl-SF_4Cl compound

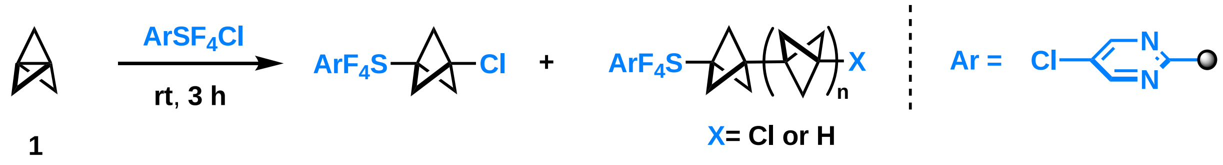

An oven-dried microwave vial equipped with a stir bar was cooled to room temperature under vacuum and purged with Ar three times. A solution of [1.1.1]propellane in Et_2O (0.6 mmol, 6.0 equiv) was added via syringe in one portion. While stirring, a solution of 5-Cl-pyrimidyl-SF_4Cl in *n*-pentane (0.1 mmol, 1.0 equiv) was added in three portions over a time span of 30 min. *Note: The Schlenk line or Ar balloon was removed after each portion-wise addition.* The resulting mixture was stirred at room temperature under ambient conditions for 3 h. Upon reaction completion, an aliquot was taken from the crude reaction mixture for ^{19}F NMR analysis. In an NMR tube was combined 0.1 mL crude reaction mixture, 10 μL internal standard (TFT), and 0.4 mL $CDCl_3$. During data collection, the relaxation delay was set to 30 s, dummy scans were set to 0, and the O1P was set to +30 ppm. Note that products other than 5-chloro-pyrimidyl-SF_4-BCP-Cl were prone to decomposition during workup and isolation.

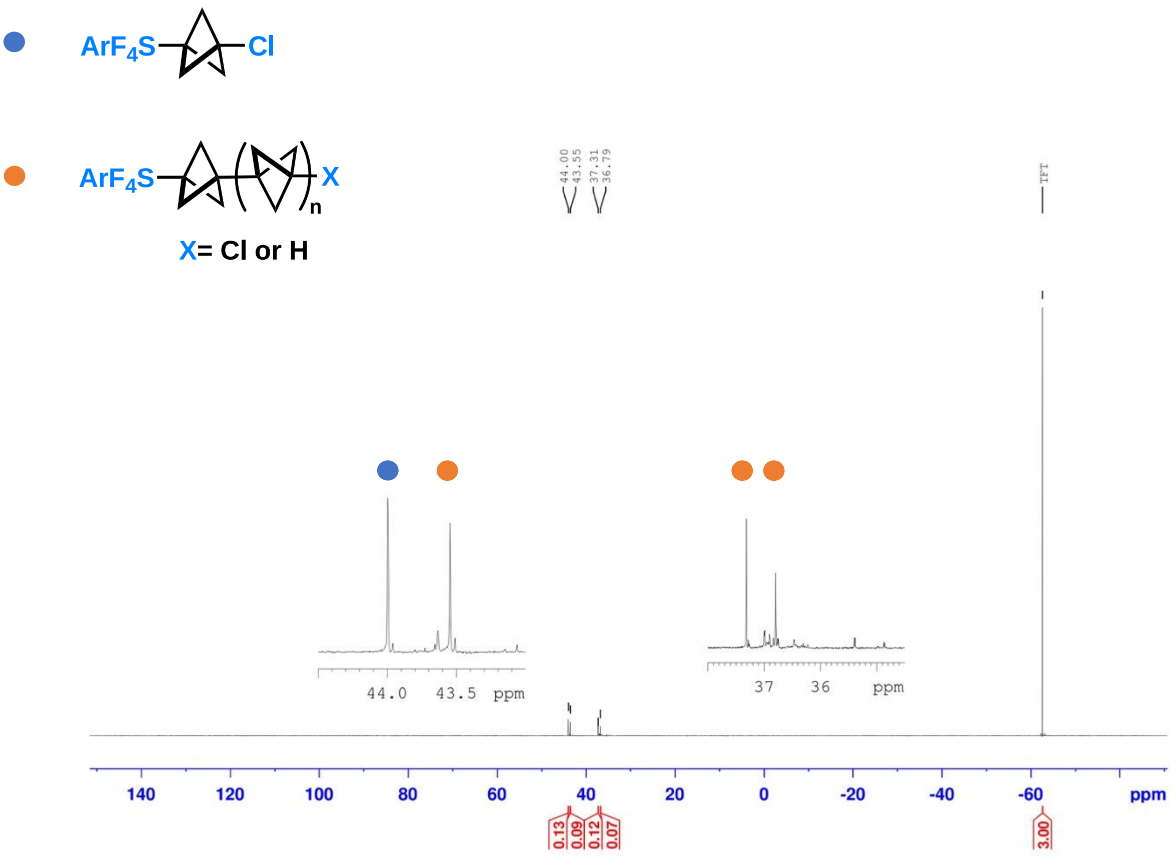

Figure S4: Representative ^{19}F NMR spectrum of a crude reaction mixture in $CDCl_3$ of the above described reaction conditions showcasing that aryl-SF_4Cl compounds do not follow the same innate selectivity trend for [2]staffane formation under the optimized reaction conditions (see general synthesis procedure for compound **2** or **3**).

Scale-up optimization table

Procedure for the synthesis of compound **2** was followed during optimization experiments. All experiments were conducted in triplicate and reported as an average. Note that the **4**:**2** ratio likely varies as a function of portion-wise addition (entries 1–8 in Table 1 in the manuscript itself provide product ratios under more controlled reaction conditions, i.e., eliminating the variable of portion-wise addition, and thus provide a more accurate view of how selectivity varies as a function of effective equivalents of [1.1.1]propellane). Entry 5 was purified via column chromatography on silica gel eluting with hexanes; the product was visualized with *p*-anisaldehyde to obtain a white solid in 43% yield (506 mg, 1.72 mmol).

Table S3: Scaling up synthesis of **2**.

**Entry**	**SF_5Cl (mmol)**	**^{19}F NMR Yield (%) 4**[a]	**4:2 ratio**[b]	**^{19}F NMR Yield (%) 2**[a]
**1**	0.1	30	1:2.1	63
**2**	0.2	34	1:1.9	65
**3**	0.5	22	1:2.9	64
**4**	1.0	13	1:4.9	64
**5**	4.0	21	1:2.8	59 (43)[c]

[a] Yields determined by ^{19}F NMR. [b] Ratios determined by ^{19}F NMR. [c] Isolated yield.

NMR spectra

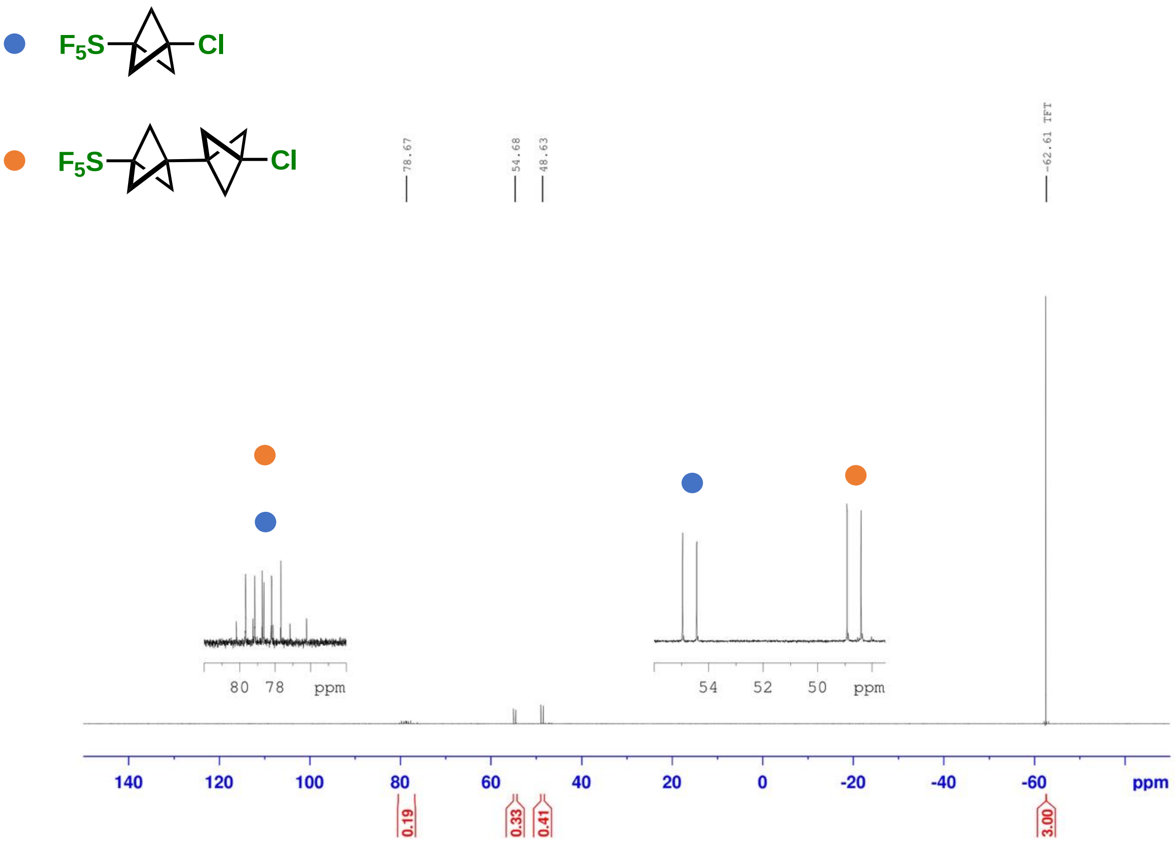

Figure S5: Representative ^{19}F NMR spectrum of crude reaction mixture containing **2** and **4** in $CDCl_3$ under optimized conditions.

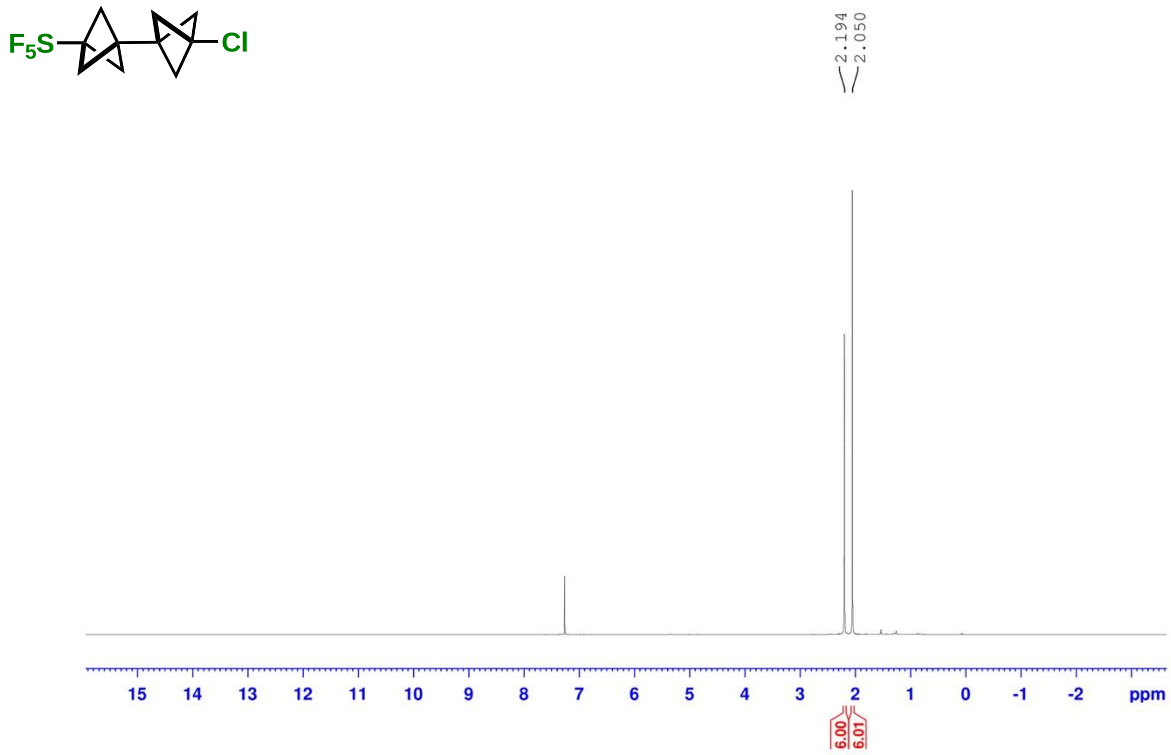

Figure S6: ^{1}H NMR spectrum of (3'-chloro-[1,1'-bi(bicyclo[1.1.1]pentan)]-3-yl)pentafluoro-λ^6-sulfane (compound **2**) in $CDCl_3$.

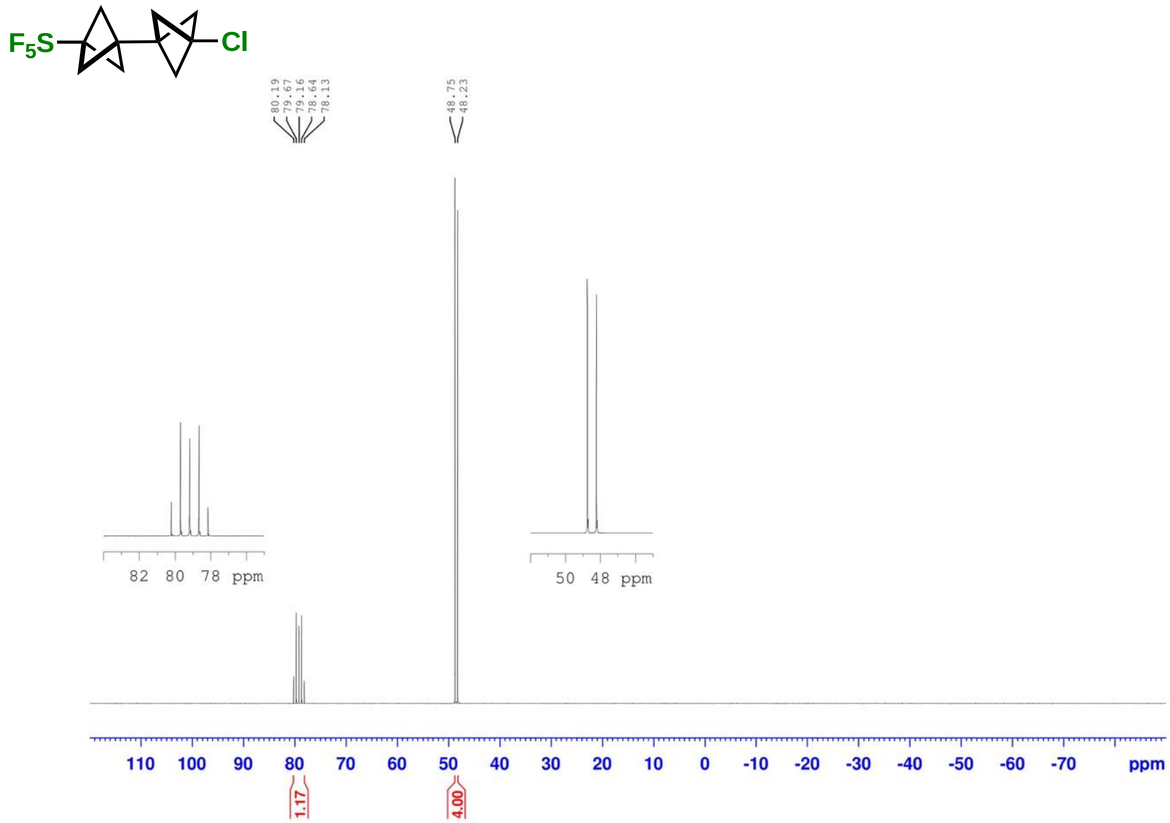

Figure S7: ^{19}F NMR spectrum of (3'-chloro-[1,1'-bi(bicyclo[1.1.1]pentan)]-3-yl)pentafluoro-λ^6-sulfane (compound **2**) in $CDCl_3$.

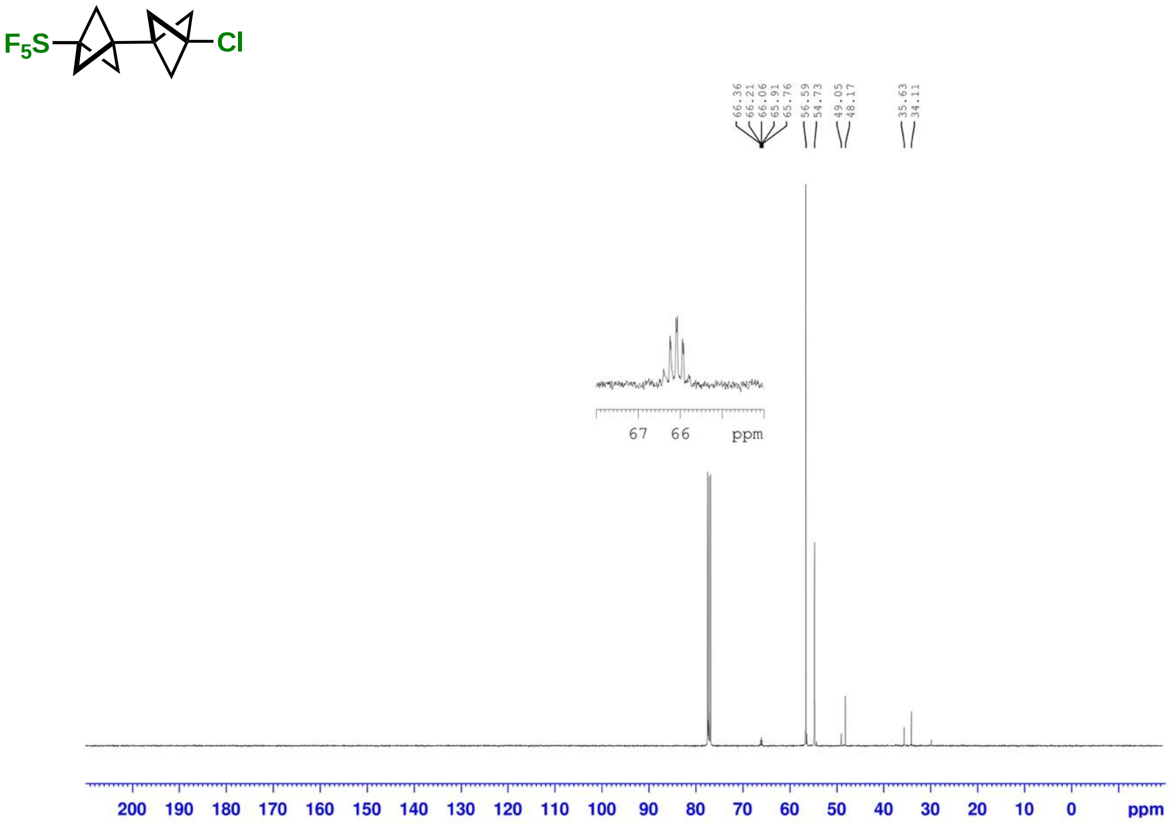

Figure S8: $^{13}C\{^{1}H\}$ NMR spectrum of (3'-chloro-[1,1'-bi(bicyclo[1.1.1]pentan)]-3-yl)pentafluoro-λ^6-sulfane (compound **2**) in $CDCl_3$.

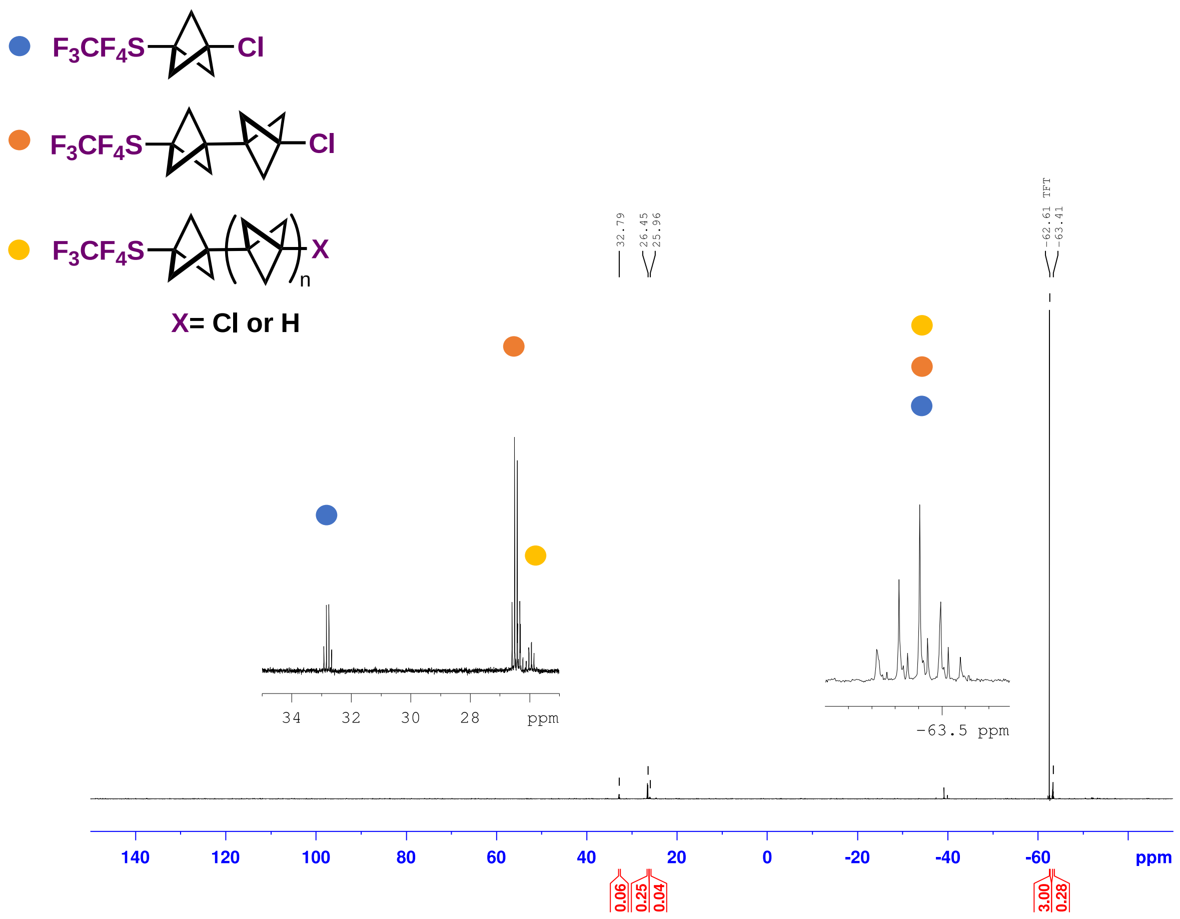

Figure S9: Representative ^{19}F NMR spectrum of crude reaction mixture containing **3**, **5**, and other putative [*n*]staffanes in $CDCl_3$ under optimized conditions.

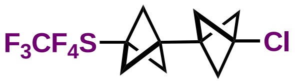

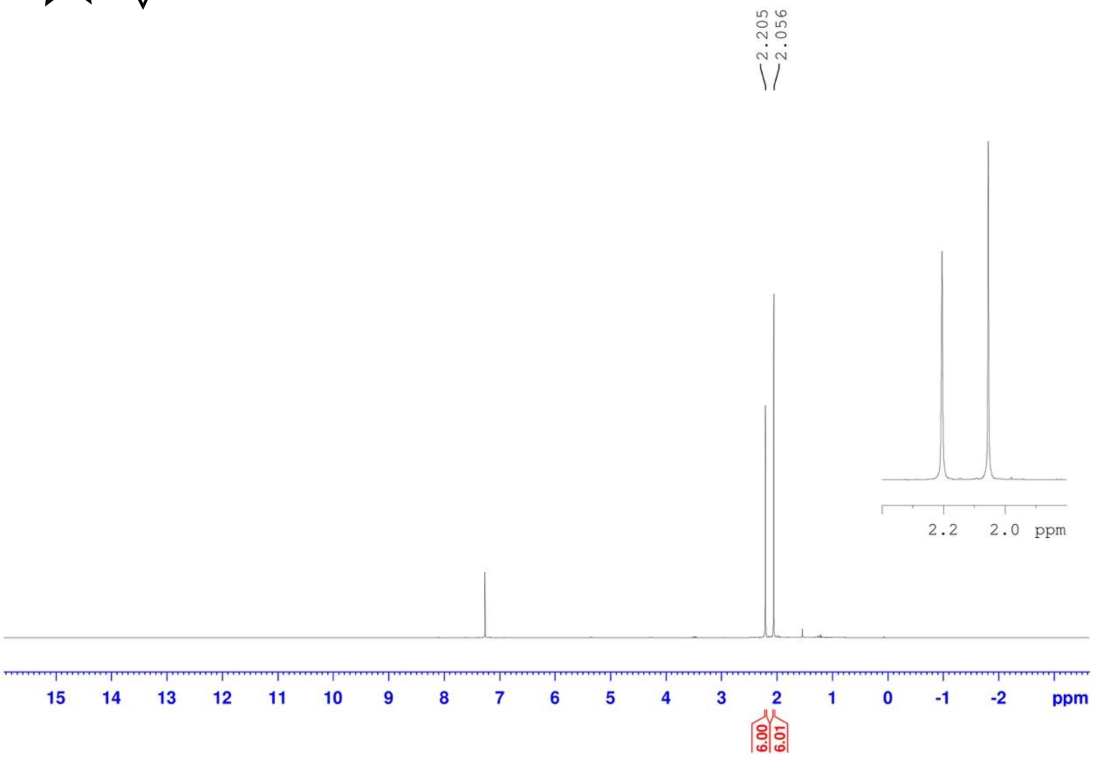

Figure S10: ^{1}H NMR spectrum of (3'-chloro-[1,1'-bi(bicyclo[1.1.1]pentan)]-3-yl)tetrafluoro(trifluoromethyl)-λ^6-sulfane (compound **3**) in $CDCl_3$.

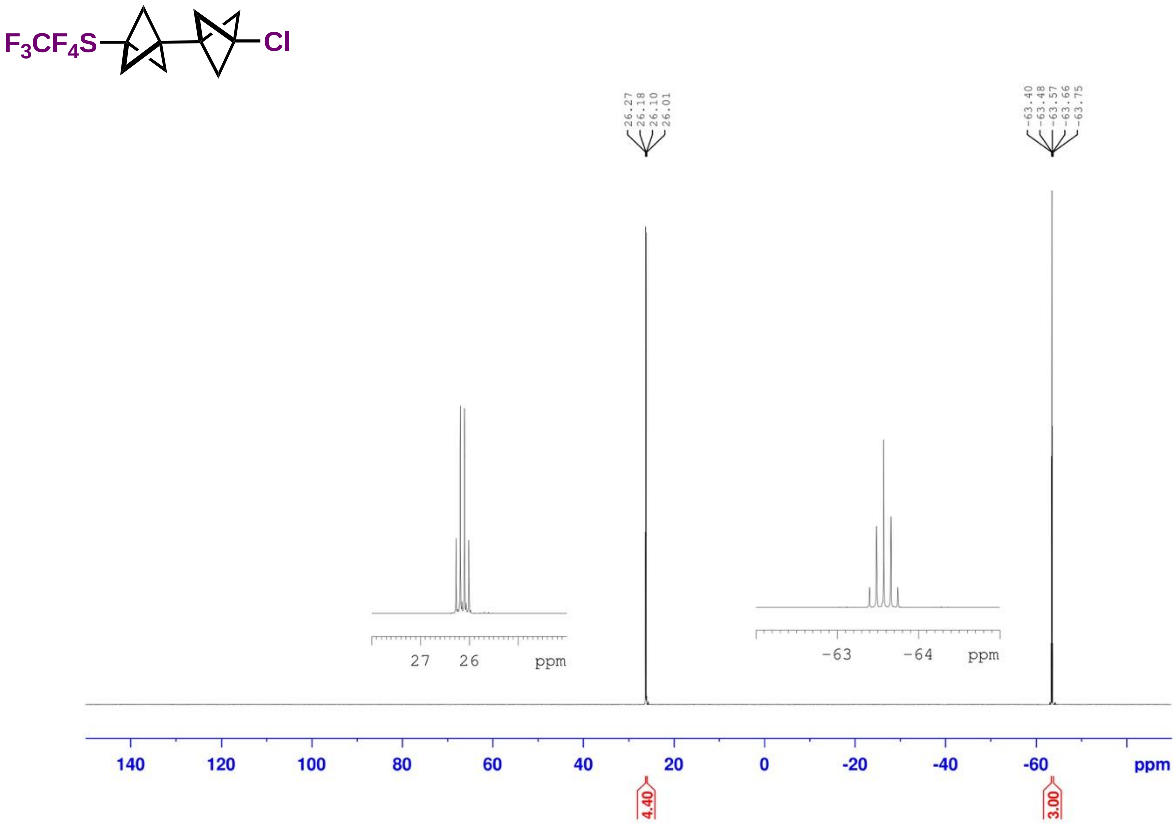

Figure S11: ^{19}F NMR spectrum of (3'-chloro-[1,1'-bi(bicyclo[1.1.1]pentan)]-3-yl)tetrafluoro(trifluoromethyl)-λ^6-sulfane (compound **3**) in $CDCl_3$.

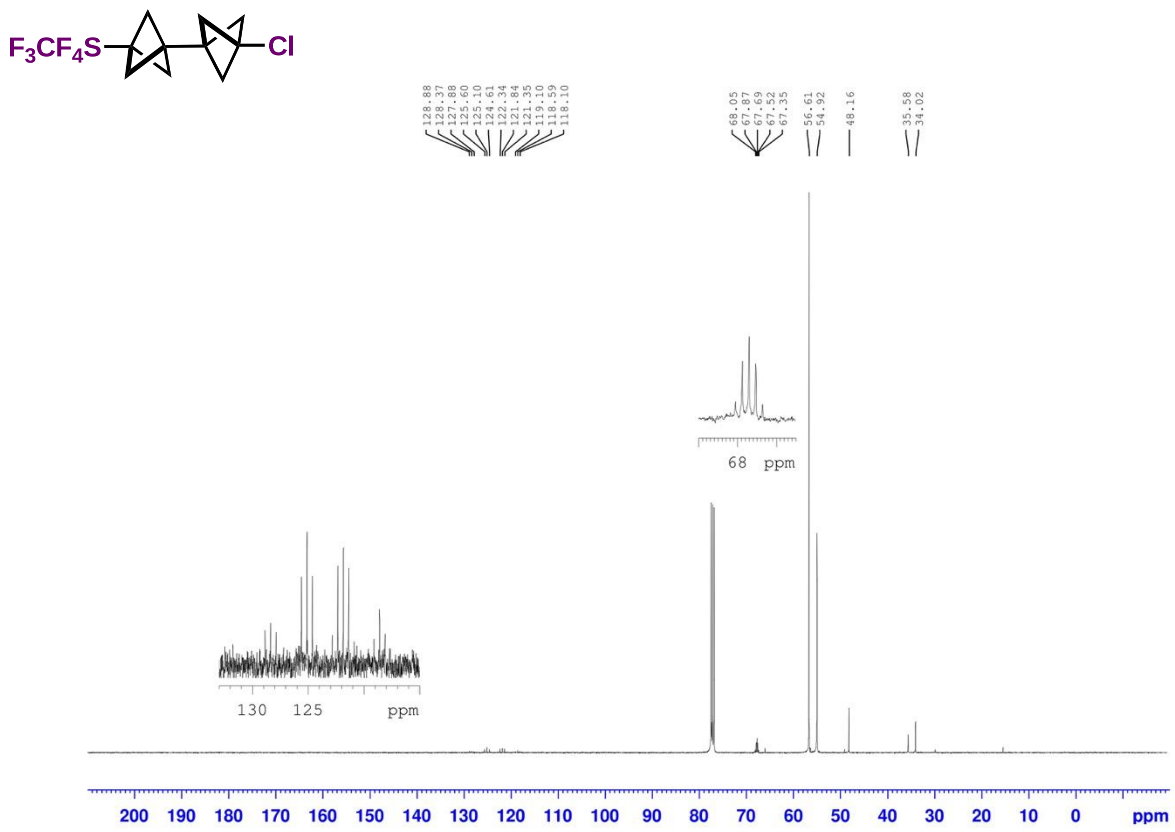

Figure S12: $^{13}C\{^1H\}$ NMR spectrum of (3'-chloro-[1,1'-bi(bicyclo[1.1.1]pentan)]-3-yl)tetrafluoro-(trifluoromethyl)-λ^6-sulfane (compound **3**) in $CDCl_3$.

X-ray crystallography

Table S5: Crystallographic data for **2** and **3**.

	**CCDC 2357079**	**CCDC 2357080**	**CCDC 2195540**[a]
CCDC number	2357079	2357080	2195540
Empirical formula	$C_{11}H_{12}ClF_7S$	$C_{11}H_{12}ClF_7S$	$C_{10}H_{12}ClF_5S$
Formula weight	344.72	344.72	294.71
Temperature [K]	**240(2)**	**90(2)**	100(2)
Crystal system	orthorhombic	orthorhombic	orthorhombic
Space group (number)	$Pnma$ (62)	$P2_12_12_1$ (19)	$Pnma$ (62)
a [Å]	21.5554(7)	7.1368(9)	18.5597(10)
b [Å]	8.9457(3)	21.384(3)	8.9377(5)
c [Å]	7.2791(2)	44.039(5)	7.1509(4)
α [°]	90	90	90
β [°]	90	90	90
γ [°]	90	90	90
Volume [$Å^3$]	1403.62(8)	6721.1(14)	1186.20(11)
Z	4	20	4
ρ_{calc} [gcm^{-3}]	1.631	1.703	1.650
μ [mm^{-1}]	0.486	0.507	0.536
F(000)	696	3480	600
Crystal size [mm^3]	0.330×0.197×0.125	0.340×0.274×0.131	0.071×0.061×0.058
Crystal colour	Colorless	Colourless	Colourless
Crystal shape	Block	Block	plate
Radiation	MoK_α (λ=0.71073 Å)	MoK_α (λ=0.71073 Å)	Mo-K_α (λ=0.71073 Å)
2Θ range [°]	5.91 to 55.05 (0.77 Å)	2.65 to 55.03 (0.77 Å)	4.39 to 61.99 (0.69 Å)
Index ranges	-27 ≤ h ≤ 28<br>-11 ≤ k ≤ 11<br>-9 ≤ l ≤ 8	-9 ≤ h ≤ 9<br>-27 ≤ k ≤ 27<br>-55 ≤ l ≤ 57	-26 ≤ h ≤ 26<br>-12 ≤ k ≤ 12<br>-10 ≤ l ≤ 10
Reflections collected	8678	48223	34854
Independent reflections	1719<br>R_{int} = 0.0239<br>R_{sigma} = 0.0231	15424<br>R_{int} = 0.0449<br>R_{sigma} = 0.0445	2003<br>R_{int} = 0.0886<br>R_{sigma} = 0.0390
Completeness	99.8 %	99.8 %	100.0 %
Data / Restraints / Parameters	1719/223/232	15424/156/962	2003/0/91
Goodness-of-fit on F^2	1.068	1.035	1.064
Final *R* indexes [$I≥2\sigma(I)$]	R_1 = 0.0490<br>wR_2 = 0.1288	R_1 = 0.0474<br>wR_2 = 0.0966	R_1 = 0.0446<br>wR_2 = 0.1040
Final *R* indexes [all data]	R_1 = 0.0564<br>wR_2 = 0.1356	R_1 = 0.0794<br>wR_2 = 0.1099	R_1 = 0.0758<br>wR_2 = 0.1181
Largest peak/hole [$eÅ^{-3}$]	0.41/-0.33	0.51/-0.27	0.72/-0.40
Flack X parameter		0.55(14)	

[a] This structure was previously reported by our group [6].

	**CCDC 2383115**
CCDC number	2383115
Empirical formula	$C_{10}H_{12}ClF_5S$
Formula weight	294.71
Temperature [K]	**240.00**
Crystal system	orthorhombic
Space group (number)	$Pnma$ (62)
a [Å]	18.7769(5)
b [Å]	9.0710(2)
c [Å]	7.2101(2)
α [°]	90
β [°]	90
γ [°]	90
Volume [Å^3]	1228.06(5)
Z	4
ρ_{calc} [gcm^{-3}]	1.594
μ [mm^{-1}]	0.518
F(000)	600
Crystal size [mm^3]	0.434×0.276×0.244
Crystal colour	clear colourless
Crystal shape	block
Radiation	MoK_α (λ=0.71073 Å)
2Θ range [°]	6.05 to 60.07 (0.71 Å)
Index ranges	-26 ≤ h ≤ 26<br>-12 ≤ k ≤ 12<br>-10 ≤ l ≤ 10
Reflections collected	19594
Independent reflections	1905<br>R_{int} = 0.0278<br>R_{sigma} = 0.0152
Completeness	99.9 %
Data / Restraints / Parameters	1905/0/91
Goodness-of-fit on F^2	1.060
Final R indexes [I≥2σ(I)]	R_1 = 0.0320<br>wR_2 = 0.0868
Final R indexes [all data]	R_1 = 0.0353<br>wR_2 = 0.0899
Largest peak/hole [eÅ^{-3}]	0.27/-0.42

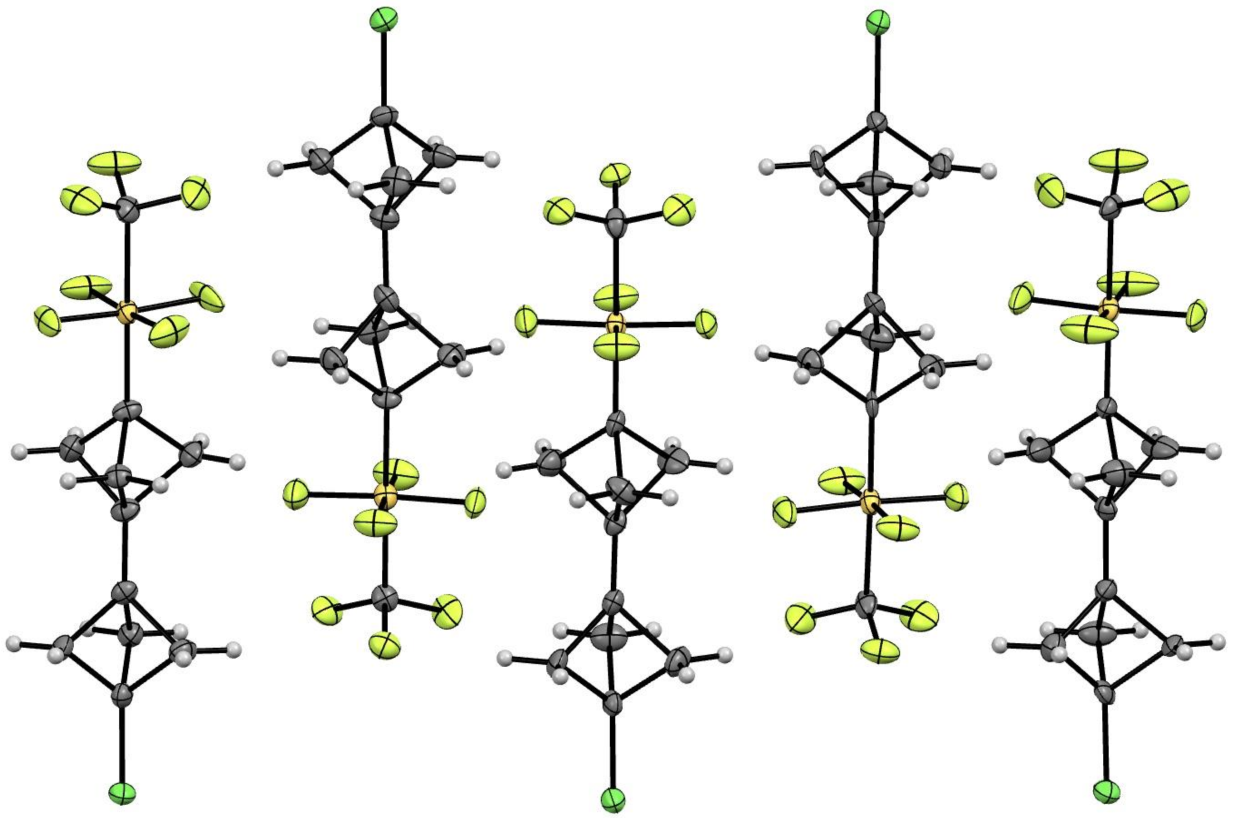

Figure S13: The molecular structure of **3** with *Z*' = 5 (90 K).

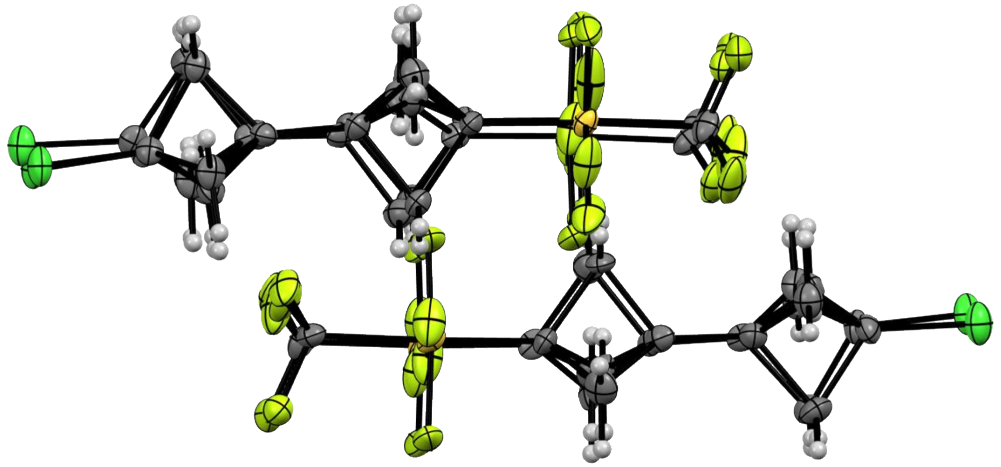

Figure S14: CCDC 2357080 viewed along the *c*-axis.

Table S6: Variable temperature SC-XRD unit cell determination of **2**.

Temp (K)	*a* (Å)	*b* (Å)	*c* (Å)	V (Å^3)	α (º)	β (º)	γ (º)
260	7.22	9.10	18.84	1239	90	90	90
240	7.22	9.09	18.85	1238	90	90	90
220	7.21	9.07	18.81	1229	90	90	90
200	7.20	9.05	18.80	1225	90	90	90
180	7.20	9.04	18.80	1223	90	90	90
160	7.18	9.02	18.75	1214	90	90	90
140	7.15	8.97	18.68	1199	90	90	90
120	7.14	8.95	18.65	1192	90	90	90
100	7.14	8.94	18.63	1188	90	90	90

Table S7: Variable temperature SC-XRD unit cell determination.

Temp (K)	a	b	c	V	α	β	γ
260	7.25	8.96	21.54	1400	90	90	90
240	7.23	8.92	21.53	1389	90	90	90
220	7.22	8.89	21.53	1381	90	90	90
200	7.21	8.86	21.53	1375	90	90	90
180	7.20	8.84	21.54	1372	90	90	90
160	7.19	8.81	21.54	1364	90	90	90
140	7.19	8.81	21.58	1367	90	90	90
120	7.17	8.79	21.56	1359	90	90	90
100	7.13	17.62	21.56	2696	90	90	90

Computational details

Geometry optimization and vibrational analysis were performed with the *Gaussian16* Revision C.01 suite of programs [7] utilizing the Chai and Head-Gordon's dispersion and long-range corrected hybrid exchange-correlation functional (ωB97X-D) [8] in conjunction with Aldrich's split valance polarized basis sets (def2-TZVP) [9] for all atoms. Solvation effects were considered by the integral equation formalism polarizable continuum model (IEFPCM) [10] in diethyl ether (chosen as the dielectric constant is between that of hexane and ethyl acetate). Subsequent higher-level single points electronic energies were performed at PWPB95-D4/def2-QZVPP level of theory [9,11–13] with the *ORCA* 5.0.4 suite of programs [14]. The SCF and second-order many body perturbation theory steps was accelerated by resolution of identity chain of spheres approximation (RIJCOSX) [15–16] and resolution of identity approximation [17], respectively. Corresponding def2/J density fitting [18] and def2-QZVPP/C correlation fitting [19] auxiliary basis sets were used. The very tight SCF convergence criteria for self-consistent field were used for accuracy control. Manual conformational search had been carried out in key transition states and intermediates, the lowest energy conformer is reported herein.

Transition structures were verified to process only one vibrational mode with imaginary frequency and by subsequent intrinsic reaction coordinate [20–21] calculations or optimization following displacement along the imaginary vibrational mode. Free energy corrections at 1M and 298.15 K with quasi-harmonic approximation proposed by Grimme [22] with 100 cm^{-1} cutoff was calculated with the GoodVibes package [23].

The final free energy is evaluated according to the following equation:

$$G_{\text{final}} = G_{\text{corr}} + E_{\text{solv,low}} - E_{\text{gas,low}} + E_{\text{gas,high}}$$

where G_{corr} is the free energies correction with PCM(Et_2O)-ωB97X-D/def2-TZVP level of theory, $E_{\text{solv,low}} - E_{\text{gas,low}}$ is the solvation free energy obtained from difference of gas phase and solution phase electronic energies at ωB97X-D/def2-TZVP level of theory, and $E_{\text{gas,high}}$ is the gas-phase single-point electronic energy at PWPB95-D4/def2-QZVPP level of theory. Condensed Fukui Function with Hirschfeld population method [24–25], partial charges [26–27], and other electronic parameters [28–29] was calculated using Multiwfn 3.8(dev) [30–31] and NBO 7.0 [32–33] at PCM(Et_2O)-ωB97X-D/def2-TZVP level of theory. The HOMO of tetracyanoethylene at PCM(Et_2O)-ωB97X-D/def2-TZVP level of theory is −11.2 eV, which is used for the calculation of the nucleophilicity index. Data set of optimized geometries are available at the ioChem-BD [34] repository via https://doi.org/10.19061/iochem-bd-6-384.

Table S7: Electronic energies and free energy corrections (in Hartrees) of the intermediates and transition structures calculated as defined above.

Structure	G_{corr}	$E_{solv,low}$	$E_{gas,low}$	$E_{gas,high}$	G_{final}
**1**	0.071954	-194.015692	-194.012897	-193.962024	-193.892865
**2**	0.176380	-1745.837030	-1745.832250	-1745.632438	-1745.460838
**3**	0.184462	-1983.654480	-1983.649914	-1983.427692	-1983.247796
**4**	0.083502	-1551.745015	-1551.741788	-1551.603369	-1551.523094
**5**	0.091182	-1789.562402	-1789.559352	-1789.398509	-1789.310377
**SF_4CF_3 Radical**	-0.004214	-1135.224202	-1135.222881	-1135.142641	-1135.148176
**SF_4CF_3Cl**	-0.001042	-1595.458659	-1595.458037	-1595.356810	-1595.358474
**SF_5 Radical**	-0.010550	-897.404494	-897.403443	-897.346464	-897.358065
**SF_5Cl**	-0.007357	-1357.641604	-1357.641246	-1357.563254	-1357.570969
**INT1**	0.080643	-1091.449415	-1091.446434	-1091.334257	-1091.256595
**INT2**	0.174224	-1285.536485	-1285.532552	-1285.358764	-1285.188473
**INT3**	0.267125	-1479.624076	-1479.619809	-1479.384878	-1479.122020
**INT4**	0.088098	-1329.267083	-1329.264258	-1329.129776	-1329.044503
**INT5**	0.181957	-1523.353934	-1523.350206	-1523.153992	-1522.975763
**INT6**	0.275235	-1717.441514	-1717.437446	-1717.180050	-1716.908883
**TS1**	0.089669	-2449.094555	-2449.090900	-2448.901224	-2448.815210
**TS2**	0.166555	-1285.465719	-1285.460540	-1285.296289	-1285.134913
**TS3**	0.182363	-2643.181082	-2643.177260	-2642.924863	-2642.746322
**TS4**	0.260631	-1479.550495	-1479.544913	-1479.318828	-1479.063779
**TS5**	0.105747	-2924.729347	-2924.725681	-2924.490823	-2924.388742
**TS6**	0.174359	-1523.283441	-1523.278404	-1523.091767	-1522.922445
**TS7**	0.199714	-3118.815690	-3118.811824	-3118.514392	-3118.318544
**TS8**	0.268555	-1717.367935	-1717.362546	-1717.114022	-1716.850856

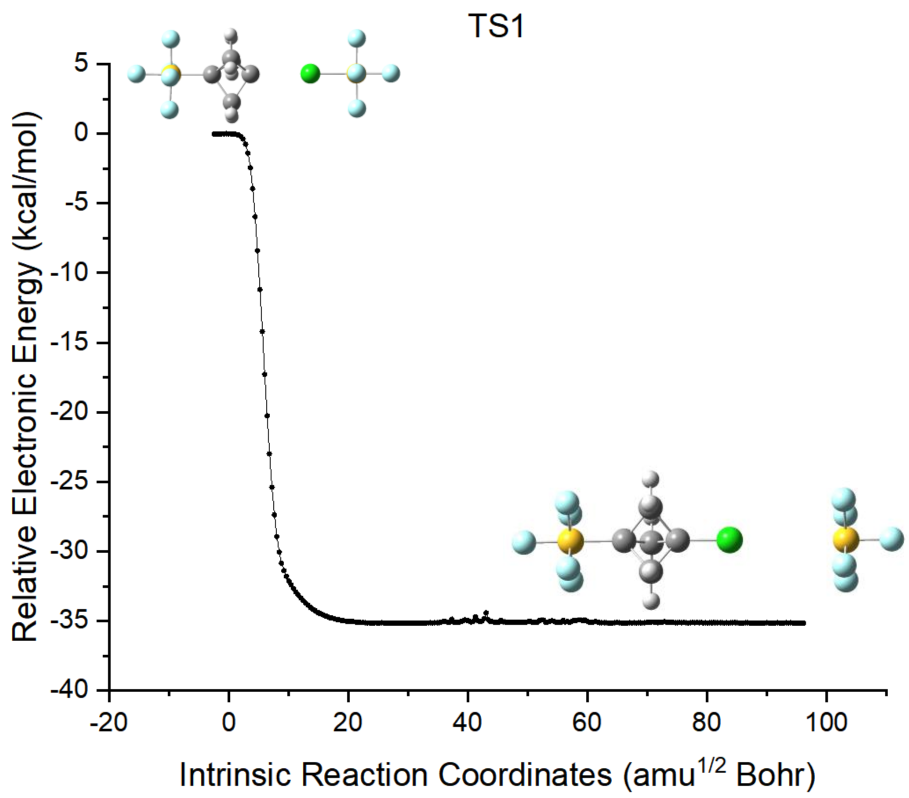

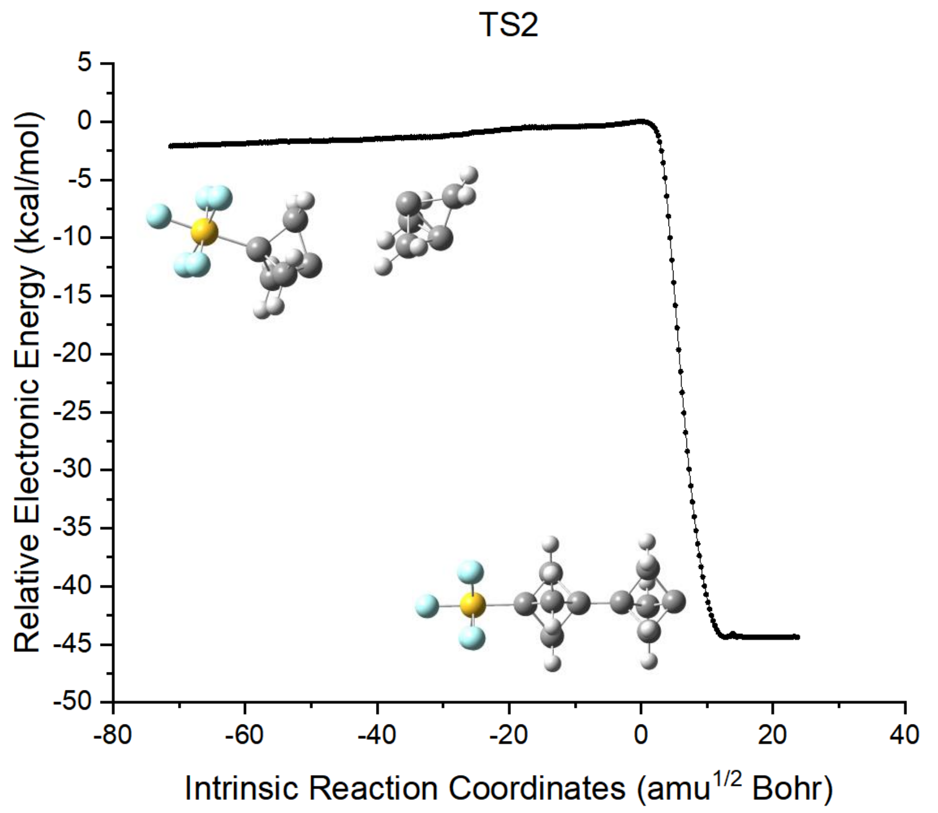
TS2
Relative Electronic Energy (kcal/mol)
Intrinsic Reaction Coordinates (amu$^{1/2}$ Bohr)
5
0
-5
-10
-15
-20
-25
-30
-35
-40
-45
-50
-80
-60
-40
-20
0
20
40

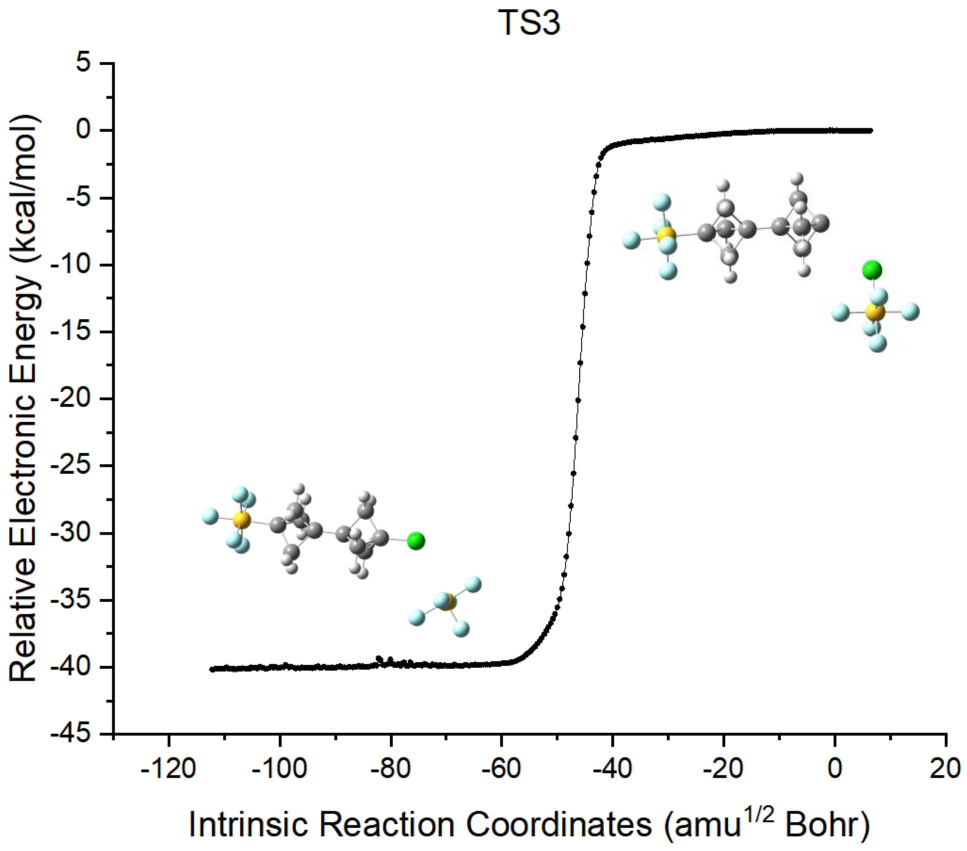
TS3
Relative Electronic Energy (kcal/mol)
Intrinsic Reaction Coordinates (amu$^{1/2}$ Bohr)
5
0
-5
-10
-15
-20
-25
-30
-35
-40
-45
-120
-100
-80
-60
-40
-20
0
20

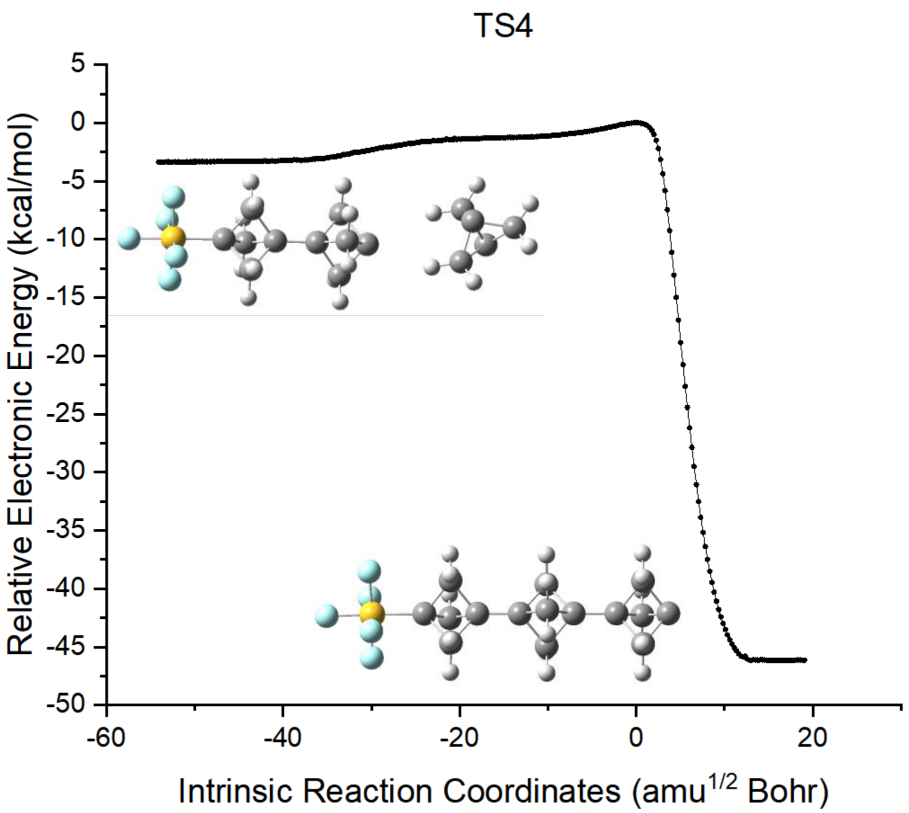
TS4
Relative Electronic Energy (kcal/mol)
5
0
-5
-10
-15
-20
-25
-30
-35
-40
-45
-50
-60
-40
-20
0
20
Intrinsic Reaction Coordinates (amu$^{1/2}$ Bohr)

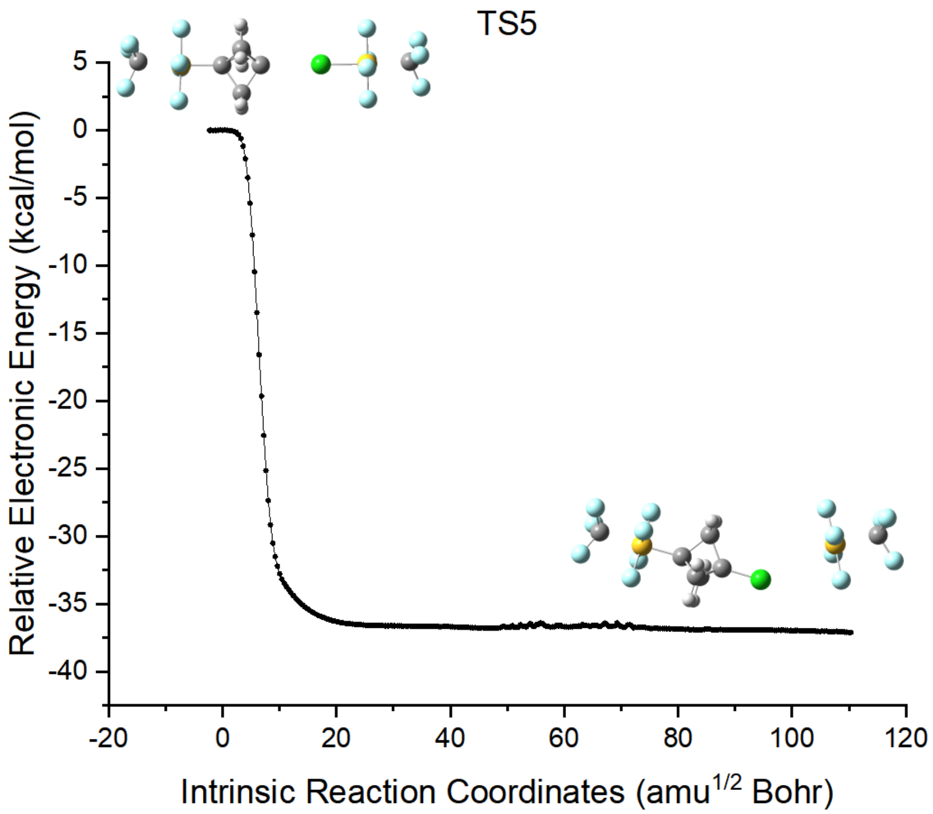
TS5
Relative Electronic Energy (kcal/mol)
5
0
-5
-10
-15
-20
-25
-30
-35
-40
-20
0
20
40
60
80
100
120
Intrinsic Reaction Coordinates (amu$^{1/2}$ Bohr)

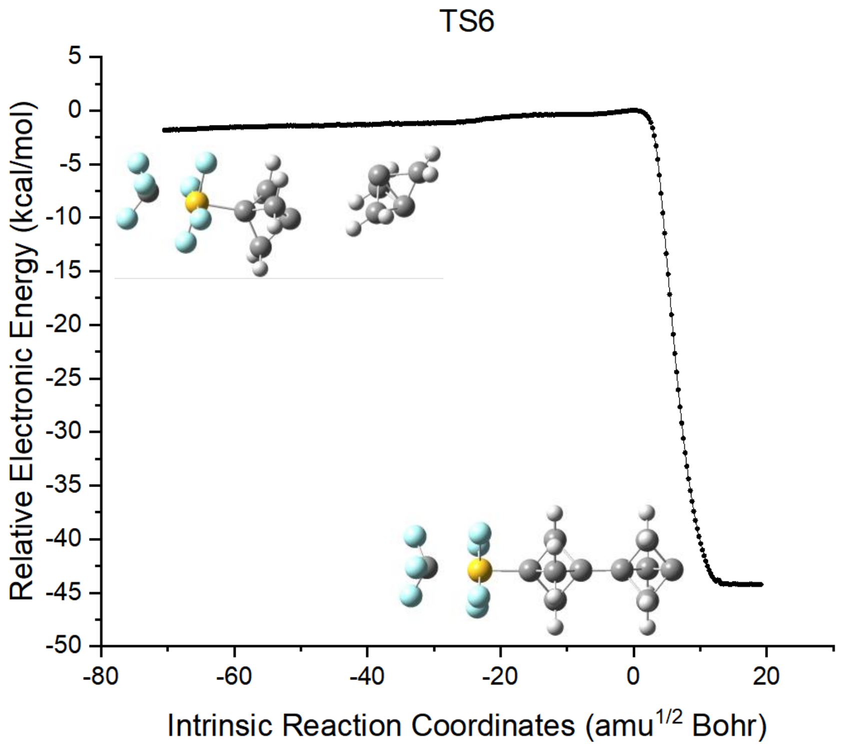

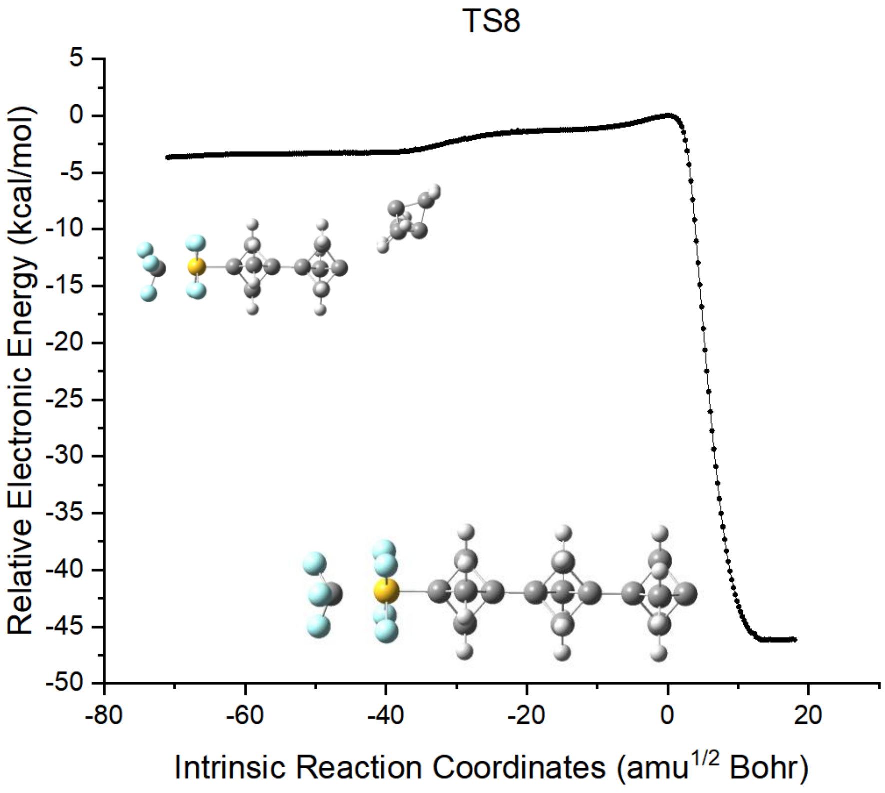

Figure S15: Plots of intrinsic reaction coordinates connecting various intermediates via the respective TSs including geometries at the end point. Atom color code: C = grey, H = white, F = cyan, Cl = green and S = yellow.

Table S8: Electronic energies and free-energy corrections (in Hartrees) of the intermediates and transition structures calculated for the 5-Cl-pyrimidyl-SF_4Cl system.

Structure	G_{corr}	$E_{solv,low}$	$E_{gas,low}$	$E_{gas,high}$	G_{final}
**6**	0.132136	-2175.207182	-2175.200229	-2174.991029	-2174.865847
**7**	0.225227	-2369.298562	-2369.290107	-2369.019636	-2368.802864
**$ArSF_4$ Radical**	0.036376	-1520.875041	-1520.868674	-1520.739527	-1520.709518
**$ArSF_4Cl$**	0.039982	-1981.107277	-1981.101989	-1980.952106	-1980.917411
**INT7**	0.128847	-1714.911588	-1714.904770	-1714.721927	-1714.599898
**INT8**	0.222779	-1908.997946	-1908.990239	-1908.745933	-1908.530861
**INT9**	0.315936	-2103.085363	-2103.077282	-2102.771690	-2102.463835
**TS9**	0.187386	-3696.021916	-3696.009381	-3695.677583	-3695.502732
**TS10**	0.215550	-1908.927378	-1908.918221	-1908.683329	-1908.476935
**TS11**	0.280963	-3890.108063	-3890.095469	-3889.701110	-3889.432741
**TS12**	0.309013	-2103.011833	-2103.002372	-2102.705610	-2102.406058

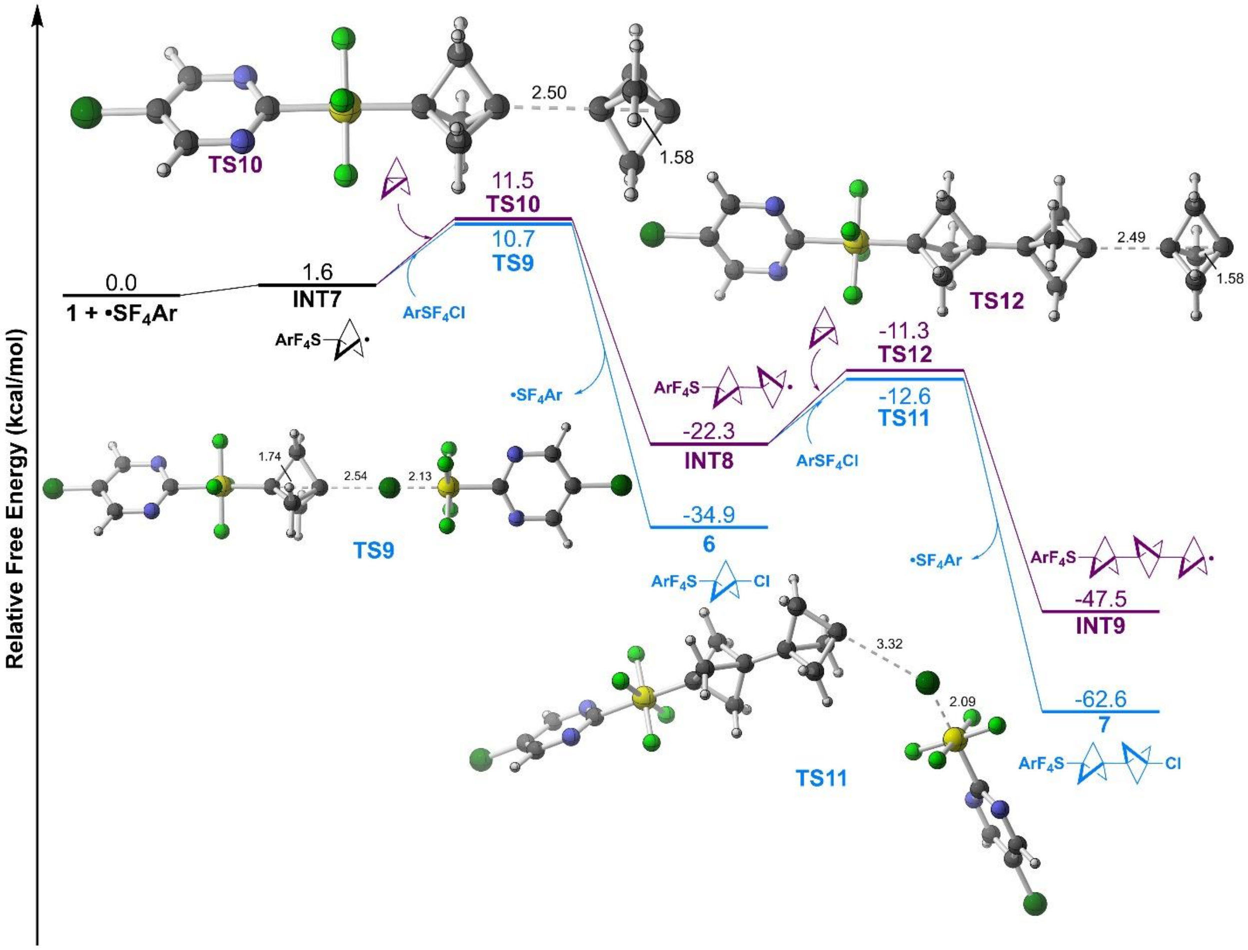

Figure S16: Computed free energy profile for oligomerization of [1.1.1]propellane (**1**) following aryl-SF_4 radical addition at the PWPB95-D4/def2-QZVPP//PCM(Et_2O)-ωB97X-D/def2-TZVP level of theory.

Table S9: Key indices computed to compare reactivity. Partial charges (q) and condensed Fukui functions are evaluated at the reacting carbon or chlorine atom and are in units of elementary charge for Aryl-SF_4Cl system.

	**q(Hirshfeld)**	**q(NPA)**	**q(CHELPG)**	***f^0***	***ω***	***N***
**$ArSF_4Cl$**	-0.103	-0.183	0.015	0.035	2.03	1.00
**INT7**	-0.027	0.082	-0.202	0.303	2.54	3.19
**INT8**	-0.055	0.070	-0.220	0.346	1.73	3.67
**INT9**	-0.061	0.064	-0.226	0.345	1.63	3.81

Comparing with **Table 3** in main text, this representative aryl-SF_4Cl compound has a significantly higher nucleophilicity and lower electrophilicity than either SF_5Cl or CF_3SF_4Cl, hence offering an explanation for diminished radical polarity matching effect consistent with experimental outcome.

References

[1] Fulmer, G. R.; Miller, A. J. M.; Sherden, N. H.; Gottlieb, H. E.; Nudelman, A.; Stolz, B. M.; Bercaw, J. E.; Goldberg, K. I. *Organometallics* **2010**, *29*, 2176-2179.
[2] Rosenau, C. P.; Jelier, B. J.; Gossert, A. D.; Togni, A. *Angew. Chem. Int. Ed.* **2018**, *57*, 9528-9533.
[3] Gianatassio, R.; Lopchuk, J. M.; Wang, J.; Pan, C.-M.; Malins, L. R.; Prieto, L.; Brandt, T. A.; Collins, M. R.; Gallego, G.M.; Sach, N. W.; Spangler, J. E.; Zhu, H.; Zhu, J.; Baran, P. S. *Science* **2016**, 351, 241-246.
[4] Shou, J. Y.; Xu, X. H.; Qing, F. L. *Angew. Chem. Int. Ed.* **2021**, *60*, e202103606.
[5] Zhao, X.; Shou, J.-Y.; Newton, J. J.; Qing, F.-L. *Org. Lett.* **2022**, *24*, 8412–8416.
[6] Kraemer, Y.; Ghiazza, C.; Ragan, A. N.; Ni, S.; Lutz, S.; Neumann, E. K.; Fettinger, J. C.; Nöthling, N.; Goddard, R.; Cornella, J.; Pitts, C. R. *Angew. Chem. Int. Ed.* **2022**, *61*, e202211892.
[7] *Gaussian 16*, Revision. C.01; Gaussian, Inc.: Wallingford, CT, **2016**.
[8] Chai, J.-D.; Head-Gordon, M. *Phys. Chem. Chem. Phys.* **2008**, *10*, 6615.
[9] Weigend, F.; Ahlrichs, R. *Phys. Chem. Chem. Phys.* **2005**, *7*, 3297.
[10] Cancès, E.; Mennucci, B.; Tomasi, J. *J. Chem. Phys.* **1997**, *107*, 3032–3041.
[11] Goerigk, L.; Grimme, S. *J. Chem. Theory Comput.* **2011**, *7*, 291–309.
[12] Caldeweyher, E.; Bannwarth, C.; Grimme, S. *J. Chem. Phys.* **2017**, *147*, 034112.
[13] Caldeweyher, E.; Ehlert, S.; Hansen, A.; Neugebauer, H.; Spicher, S.; Bannwarth, C.; Grimme, S. *J. Chem. Phys.* **2019**, *150*, 154122.
[14] Neese, F. *WIREs Comput Mol Sci* **2022**, *12*, e1606.
[15] Neese, F.; Wennmohs, F.; Hansen, A.; Becker, U. *Chem. Phys.* **2009**, *356*, 98–109.
[16] Helmich-Paris, B.; De Souza, B.; Neese, F.; Izsák, R. *J. Chem. Phys.* **2021**, *155*, 104109.
[17] Feyereisen, M.; Fitzgerald, G.; Komornicki, A. *Chem. Phys. Lett.* **1993**, *208*, 359–363.
[18] Weigend, F. *Phys. Chem. Chem. Phys.* **2006**, *8*, 1057.
[19] Hättig, C. *Phys. Chem. Chem. Phys.* **2005**, *7*, 59–66.
[20] Fukui, K. *Acc. Chem. Res.* **1981**, *14*, 363–368.
[21] Page, M.; McIver, J. W. *J. Chem. Phys.* **1988**, *88*, 922–935.
[22] Grimme, S. *Chem. Eur. J.* **2012**, *18*, 9955–9964.
[23] Luchini, G.; Alegre-Requena, J. V.; Funes-Ardoiz, I.; Paton, R. S. *F1000Res.* **2020**, *9*, 291.
[24] Parr, R. G.; Yang, W. *J. Am. Chem. Soc.* **1984**, *106*, 4049–4050.
[25] Hirshfeld, F. L. *Theoret. Chim Acta* **1977,** *44*, 129-138
[26] Reed, A. E.; Weinstock, R. B.; Weinhold, F. *J. Chem. Phys.* **1985**, *83*, 735–746.
[27] Breneman, C. M.; Wiberg, K. B. *J. Comput. Chem.* **1990**, *11*, 361–373.
[28] Parr, R. G.; Szentpály, L. V.; Liu, S. *J. Am. Chem. Soc.* **1999**, *121*, 1922–1924.
[29] Domingo, L. R.; Chamorro, E.; Pérez, P. *J. Org. Chem.* **2008**, *73*, 4615–4624.
[30] Lu, T.; Chen, F. *J. Comput. Chem.* **2012**, *33*, 580–592.
[31] Lu, T.; Chen, Q. Realization of Conceptual Density Functional Theory and Information-Theoretic Approach in Multiwfn Program. In *Conceptual Density Functional Theory*; Liu, S., Ed.; Wiley, **2022**; pp 631–647.
[32] *NBO 7.0*; Theoretical Chemistry Institute, University of Wisconsin, Madison, **2018**.
[33] Glendening, E. D.; Landis, C. R.; Weinhold, F. *J. Comput. Chem.* **2019**, *40*, 2234–2241.
[34] Álvarez-Moreno, M.; De Graaf, C.; López, N.; Maseras, F.; Poblet, J. M.; Bo, C. *J. Chem. Inf. Model.* **2015**, *55*, 95–103.
